# Real-time tracking of brain oxygen gradients and blood flow during functional activation

**DOI:** 10.1117/1.NPh.9.4.045006

**Published:** 2022-11-28

**Authors:** Sang Hoon Chong, Yi Hong Ong, Mirna El Khatib, Srinivasa Rao Allu, Ashwin B. Parthasarathy, Joel H. Greenberg, Arjun G. Yodh, Sergei A. Vinogradov

**Affiliations:** a University of Pennsylvania, Department of Physics and Astronomy, Philadelphia, Pennsylvania, United States; b University of Pennsylvania, Department of Radiation Oncology, Philadelphia, Pennsylvania, United States; c University of Pennsylvania, Perelman School of Medicine, Department of Biochemistry and Biophysics, Philadelphia, Pennsylvania, United States; d University of Pennsylvania, School of Arts and Sciences, Department of Chemistry, Philadelphia, Pennsylvania, United States; e University of South Florida, Department of Electrical Engineering, Tampa, Florida, United States; f University of Pennsylvania, Department of Neurology, Philadelphia, Pennsylvania, United States

**Keywords:** oxygen, cerebral metabolic rate of oxygen, phosphorescence quenching, Oxyphor, laser speckle contrast imaging, blood flow

## Abstract

**Significance:**

Cerebral metabolic rate of oxygen (${\mathrm{CMRO}}_{2}$) consumption is a key physiological variable that characterizes brain metabolism in a steady state and during functional activation.

**Aim:**

We aim to develop a minimally invasive optical technique for real-time measurement of ${\mathrm{CMRO}}_{2}$ concurrently with cerebral blood flow (CBF).

**Approach:**

We used a pair of macromolecular phosphorescent probes with nonoverlapping optical spectra, which were localized in the intra- and extravascular compartments of the brain tissue, thus providing a readout of oxygen gradients between these two compartments. In parallel, we measured CBF using laser speckle contrast imaging.

**Results:**

The method enables computation and tracking of ${\mathrm{CMRO}}_{2}$ during functional activation with high temporal resolution (∼7  Hz). In contrast to other approaches, our assessment of ${\mathrm{CMRO}}_{2}$ does not require measurements of CBF or hemoglobin oxygen saturation.

**Conclusions:**

The independent records of intravascular and extravascular partial pressures of oxygen, CBF, and ${\mathrm{CMRO}}_{2}$ provide information about the physiological events that accompany neuronal activation, creating opportunities for dynamic quantification of brain metabolism.

## 1 Introduction

Oxidative phosphorylation is the primary metabolic pathway by which the brain generates energy, and it requires uninterrupted delivery of oxygen to tissue.^1^ Oxygen is carried by blood and is distributed via a network of vessels, from which oxygen molecules diffuse along a concentration gradient set by the rate of oxygen consumption, known as the cerebral metabolic rate of oxygen (${\mathrm{CMRO}}_{2}$). Because there is minimal buffering of oxygen between the blood and respiring cells, cerebral blood flow (CBF) must rapidly respond to changes in neuronal activity. The oxygen concentration gradients are reflected by the vascular versus tissue (mitochondrial) partial pressures of oxygen (${\mathrm{pO}}_{2}$); thus, they encode information about oxygen consumption and supply as well as information about changes in ${\mathrm{CMRO}}_{2}$ and vascular responses.

Quantification of ${\mathrm{CMRO}}_{2}$ has been a longstanding goal in neuroscience. In a steady state, ${\mathrm{CMRO}}_{2}$ can be a useful marker of tissue pathology, such as in stroke,^2^ traumatic brain injury,^3^ and cancer.^4^ By contrast, dynamic measurements of ${\mathrm{CMRO}}_{2}$ during neuronal activation provide information about the brain metabolic events that underlie functional responses.^5^ Positron emission tomography (PET) with O15-labeled compounds is arguably the most established method for ${\mathrm{CMRO}}_{2}$ measurements in a steady-state.^6^ PET can be used in humans, but quantitative PET requires independent measurements of CBF and the total cerebral blood volume (CBV), and it relies on complex multiparametric models for the calculation of ${\mathrm{CMRO}}_{2}$.^7^ Furthermore, the temporal resolution of PET is insufficient for tracking metabolic dynamics during neuronal activation.^8^

For dynamic tracking of brain responses, the most widely used techniques are blood oxygen level-dependent functional magnetic resonance imaging (BOLD fMRI)^9^ and functional near infrared spectroscopy (fNIRS).^10^^,^^11^ In both methods, ${\mathrm{CMRO}}_{2}$ is derived from hemoglobin oxygen saturation, and its quantification requires independent measurements of oxygen extraction fraction, CBF, and CBV.^12^^–^^16^ As a result, BOLD fMRI and fNIRS are best suited for probing relative changes in ${\mathrm{CMRO}}_{2}$, and the resultant ${\mathrm{CMRO}}_{2}$ dynamics are inherently tied to the dynamics of CBF. However, ${\mathrm{CMRO}}_{2}$ and CBF may not change in the same way in response to activation, and their timing carries valuable information about the dynamics of neurovascular coupling^17^ that is inaccessible to BOLD fMRI and fNIRS.

${\mathrm{CMRO}}_{2}$ can also be inferred from measurements of lateral gradients of oxygen around individual vessels coupled with diffusion-based models, such as the Krogh-Erlang cylinder model.^18^ ${\mathrm{pO}}_{2}$ gradients around vessels were first measured with oxygen microelectrodes.^19^ More recently, two-photon phosphorescence lifetime microscopy (2PLM)^20^ has proven superior for spatially resolved ${\mathrm{pO}}_{2}$ measurements. The 2PLM method is minimally invasive and capable of probing ${\mathrm{pO}}_{2}$ at multiple locations near vessels in the brain.^21^^,^^22^ However, oxygen mapping by 2PLM requires long observation times, and hence it is currently used only for gradient measurements in steady-states.^23^^,^^24^ Nevertheless, as per this work, phosphorescence lifetime oximetry has been successfully used to measure stimulus-induced changes in brain ${\mathrm{pO}}_{2}$^25^ at speeds comparable to transients of neuronal activity. Additionally, the laser Doppler method and oxygen microelectrodes have been used concurrently during functional stimulation to track local CBF and intravascular and extravascular ${\mathrm{pO}}_{2}$ ($p_{i}O_{2}$ and $p_{e}O_{2}$) in selected locations, but ${\mathrm{CMRO}}_{2}$ was not computed.^26^

Herein, we introduce and demonstrate an all-optical approach for dynamic measurements of ${\mathrm{CMRO}}_{2}$. The new methodology is based on quantification of oxygen concentration in the brain using two macromolecular phosphorescent probes, Oxyphors PtG4 and PtR4, placed separately in the intravascular and extravascular compartments. The Oxyphors do not diffuse across the blood brain barrier and have distinguishable optical spectra. Their optical signals can be obtained independently and concurrently for direct measurement of oxygen gradients between the compartments. In our work, we sampled oxygen gradients in rat brain cortex at a rate of ∼7  Hz, but this sampling frequency can potentially be increased more than 10-fold. In parallel, we measured local CBF by laser speckle contrast imaging (LSCI). Overall, the obtained data enabled us to correlate changes in ${\mathrm{CMRO}}_{2}$ with changes in CBF and thereby ascertain the timing and relative magnitudes of the physiological events that accompany neuronal activation. The new approach is minimally invasive, offers superior time-resolution (potentially approaching milliseconds), and creates novel opportunities for dynamic tracking and quantification of absolute ${\mathrm{CMRO}}_{2}$ and neurovascular coupling dynamics.

## 2 Materials and Methods

### 2.1 Two-color Phosphorescence Lifetime Oximetry

The phosphorescent probes used in this study were Oxyphors PtG4 and PtR4. PtG4 has been used previously.^27^^–^^29^ PtR4 was synthesized specifically for this work (Supplementary Material, p. S3). PtR4 is similar to the probe PdR4,^30^ except that the central metal ion in the porphyrin is Pt(II) and the shell surrounding the porphyrin in PtR4 is composed of mixed arylglycine/glutamate dendrons. The difference between Pt and Pd porphyrin-based probes has been discussed previously.^31^ The synthesis and the photophysical data for PtR4 can be found in the Supplementary Material (pp. S3–S9). The probes were calibrated using a setup described previously.^30^

**Fig. 1:**
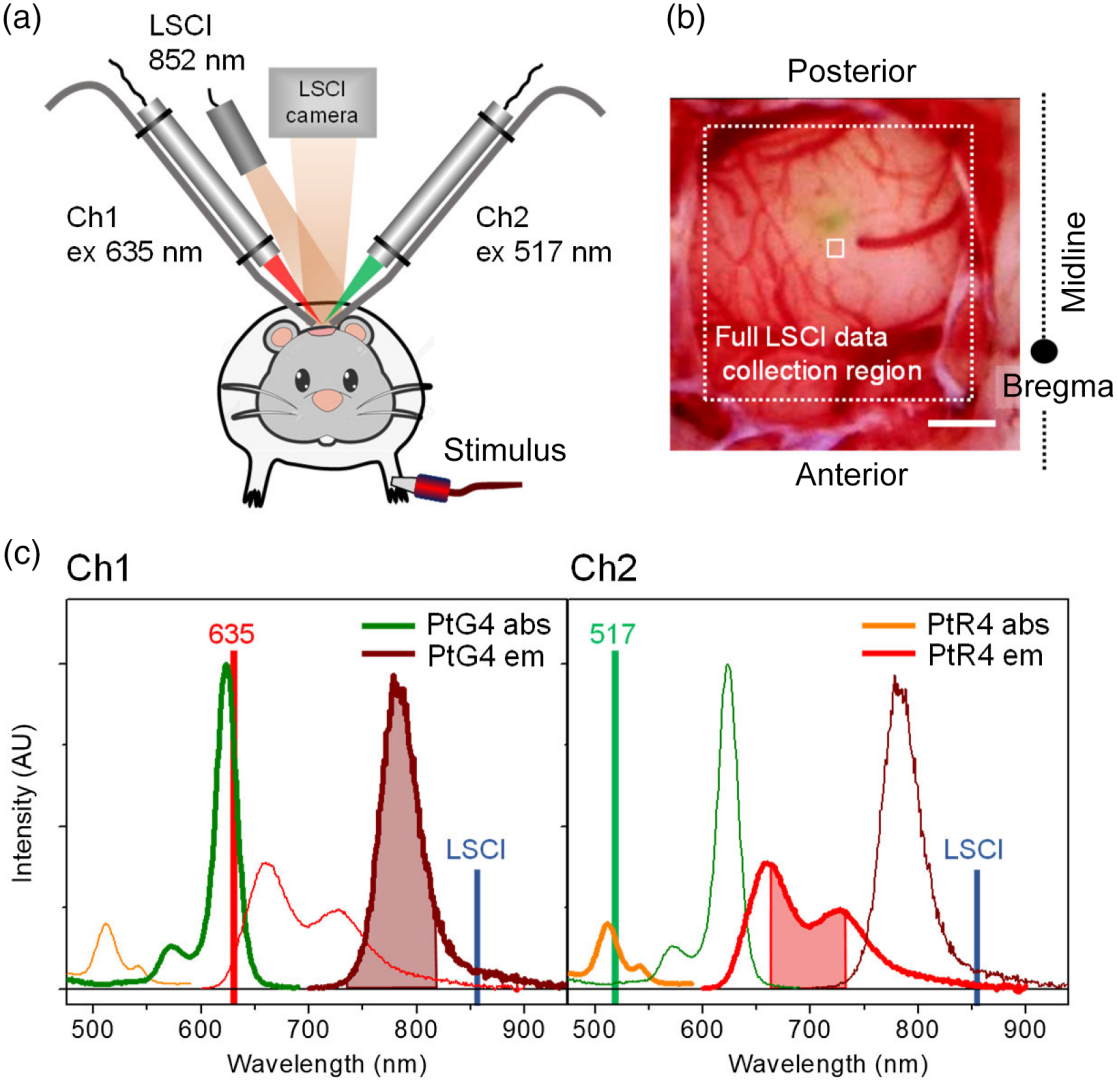
(a) Scheme of the experimental setup. (b) Representative wide-field image of the craniotomy window over the somatosensory area. Scale bar: 1 mm. The full LSCI imaging area is shown by the dashed square. The solid white square (200  μm×200  μm) indicates the region used for LSCI data processing, which coincided with the focus spot of the phosphorometer lasers (∼200  μm in diameter). This spot was located ∼300  μm away from the probe injection site (seen as a greenish area). (c) Optical absorption and phosphorescence spectra of Oxyphors PtG4 (channel 1) and PtR4 (channel 2). The laser lines (635 and 517 nm) of the respective channels and the LSCI laser line (852 nm) are shown by vertical bars. The absorption spectra are scaled by the respective extinction coefficients [$\varepsilon_{620}(\mathrm{PtG}4)\approx 1.0\times 10^{5}\;M^{-1}\;{\mathrm{cm}}^{-1}$ and $\varepsilon_{510}(\mathrm{PtR}4)\approx 2.0\times 10^{4}\;M^{-1}\;{\mathrm{cm}}^{-1}$]. The emission spectra are scaled such that their areas are proportional to the probes’ phosphorescence quantum yields ($\phi_{\mathrm{PtG}4}=0.067$ and $\phi_{\mathrm{PtR}4}=0.052$). The emission wavelength ranges isolated by the optical filters and seen by the detectors in Ch1 (PtG4) and Ch2 (PtR4) are shown by the shaded areas.

The instrument for two-color phosphorometry was constructed in-house [Fig. 1(a)]. The light sources for excitation of PtG4 and PtR4 were modulated diode lasers (Power Technology) operating at $\lambda_{\max}=630\;\mathrm{nm}$ (15 mW) and $\lambda_{\max}=517\;\mathrm{nm}$ (10 mW), respectively. Both diode lasers have a rise time of ∼50  ns. Avalanche photodiodes (APDs) were employed for light detection (C12703-01, Hamamatsu; rise time 3.3  μs). The instrument had two channels (Ch1 and Ch2) with the term channel referring to the combination of a laser, optical fiber for transmitting phosphorescence light to a detector, optical filters, and APD. The control of data acquisition was performed using a digital board (NI USB-6351, National Instruments; 1 MHz) that communicated with the host computer via a USB interface. The data acquisition and analysis software was written in C/C++ (Qt, Nokia). (See Supplementary Material, p. S8 for additional details.)

The light beams from both lasers (Ch1 and Ch2) were focused by lenses (f=60  mm and Ø=12  mm) onto the same spot (diameter∼0.2  mm) on the brain surface; the spot resided within the region of the cortex responsible for forepaw responses [Fig. 1(b)]. Phosphorescence signals were collected by single-core plastic fibers (Ø=4  mm, Fiberoptic Technology) with tips that were held ∼2  mm from the excitation spot [Fig. 1(a)]. The phosphorescence in each channel was passed through a series of optical filters and focused by a spherical lens (Ø=10  mm) onto the APD entrance aperture.

To measure ${\mathrm{pO}}_{2}$ in the intravascular and extravascular compartments, one phosphorescent probe was injected into the vasculature, and the other was injected directly into the interstitial space of the brain tissue. A single data acquisition cycle (duration T=δt+Δt) consisted of an excitation pulse (δt=10  μs), during which the laser was on, followed by the phosphorescence collection period (Δt=290  μs), during which the APD current was digitized and recorded. To increase the signal-to-noise ratio (SNR), multiple cycles (N) were executed in sequence, after which the resulting data array was transferred to the computer. The collected phosphorescence decays were averaged and analyzed by single-exponential fitting, and the phosphorescence decay time was converted to ${\mathrm{pO}}_{2}$ using a Stern–Volmer-like equation. Once a measurement in one channel was completed, it was followed immediately by measurement in the other channel. The ${\mathrm{pO}}_{2}$ gradient ($\Delta {\mathrm{pO}}_{2}$), defined here as the difference between volume-averaged $p_{i}O_{2}$ and $p_{e}O_{2}$ ($\Delta {\mathrm{pO}}_{2\;}=p_{i}O_{2}-p_{e}O_{2}$), was computed as the difference between the readings in the two channels.

For typical acquisition settings (e.g., T=300  μs and N=30), the data collection time for each channel was ∼10  ms, and the data transfer/analysis required another ∼10  ms. However, due to hardware limitations, the maximum repetition rate for two-channel acquisition was 7 Hz (∼70  ms per channel). The significant “dead time” was caused by the internal operation of the digital board, which had to undergo a reset for each measurement cycle. In the future, with more efficient hardware and optimized collection geometry, the measurement rate can be readily increased to >50  Hz.

### 2.2 Laser Speckle Contrast Imaging

The principles of LSCI have been discussed previously.^32^^,^^33^ A diode laser (852 nm, 600 mW, and LD852-SE600, Thorlabs) was positioned at a distance of ∼20  cm from the brain surface, and its beam was directed at ∼45  deg angle relative to the surface normal. The brain was imaged using a CMOS camera (acA2040-180 km, Basler AG, Germany) equipped with an infinity-corrected optical system composed of two lenses with focal lengths of f=135  mm (Mitakon Zhongyi Mark II Lens for Nikon F, Zhong Yi Optics, China) and f=75  mm (AC508-075-B, Thornlabs). Images (5-ms exposure) were acquired at a frame rate of 20 Hz using custom-written software (LabVIEW, National Instruments). The LSCI field of view (FOV) was 6.2  mm×6.2  mm; the FOV encompassed the focal spot used for the phosphorescence oximetry. LSCI images were analyzed to quantify relative changes of CBF in the brain cortex, as described elsewhere.^34^

### 2.3 Animal Preparation

Twelve adult male Sprague–Dawley rats (250 to 400 g) were used in this study. Rats were anesthetized via the inhalation of a nitrogen:oxygen (70:30) mixture containing isoflurane (∼4% for initial induction, maintained at ∼1.5%). A catheter was placed into the left femoral artery for continuous monitoring of the arterial blood pressure (ABP) (507061F pressure transducer, Harvard Apparatus). This catheter was also used for injection of the phosphorescent probe for $p_{i}O_{2}$ measurements and for injections of saline to maintain hydration. The animal’s head was fixed in a stereotaxic frame, and a 6  mm×4  mm cranial window was made in the skull over the forepaw somatosensory area [Fig. 1(b)] using a saline-cooled dental drill. The dura mater was removed, after which the exposed brain surface was continuously irrigated with artificial cerebrospinal fluid (Supplementary Material, p. S3) heated to 37°C. After the craniotomy was completed, α-chloralose was administered intraperitoneally (60  mg/kg). Simultaneously, the isoflurane dose was gradually decreased and then discontinued over a period of 30 min. Anesthesia was maintained by supplemental doses of α-chloralose (30  mg/kg,∼1  dose/hr). The depth of anesthesia was verified every 15 min by the toe-pinch method. Two electrodes for electrical stimulation were inserted subdermally into the left forepaw contralateral to the craniotomy site [Fig. 1(a)]. The distal ends of the electrodes were connected to a customizable stimulus isolator (A365, World Precision Instruments). The body temperature was monitored with a rectal probe and was maintained at 37.4°C±0.2°C using a heating pad controlled by a homeothermic monitoring system (Harvard Apparatus). The animals were under the care of the University of Pennsylvania Laboratory Animal Resources. All studies were approved by the University of Pennsylvania Institutional Animal Care and Use Committee.

### 2.4 Experimental Sequence

First, stimulation was applied to the animal’s forepaw using electrical current from a stimulus isolator (1.5 mA, 1-ms-long pulses at 3 Hz, and stimulation time 4 s), and LSCI data were collected and analyzed^34^ to locate the somatosensory cortex region within the exposed craniotomy window. Oxyphors PtR4 and PtG4 were administered as solutions in physiological saline: one intravascularly via femoral catheter (50  μL, 200  μM) and the other (0.1  μL, 300  μM) was injected directly into the brain tissue using a microinjection dispenser (Picospritzer III, Parker Hannifin Precision Fluidics Division) and a micropipette (tip diameter∼15  μm). The micropipette was inserted to a depth of 200 to 300  μm, and the Oxyphor solution was injected at a rate of ∼0.1  μL/min. Subsequently, electrical stimulation/LSCI was repeated to confirm that functional responses persisted. The laser assemblies for phosphorescence measurements were positioned above the cranial window [Fig. 1(a)], and phosphorescence signals were measured. If necessary, the amount of intravascular probe was increased by injecting additional solution through the catheter.

Each stimulation cycle was 1-min-long, and it consisted of 4-s-long baseline monitoring, followed by the application of a 4-s-long stimulus (1.5 mA and 1-ms-long pulses at 3 Hz) and a 52-s-long recovery period. Up to 15 sequential stimulation cycles were applied, during which the phosphorescence and LSCI data were recorded.

### 2.5 Modeling and Calculation of CMRO_2_

${\mathrm{CMRO}}_{2}$ was computed from the experimentally measured differences between $p_{e}O_{2}$ and $p_{i}O_{2}$ using a two-compartment tissue model published previously.^35^ In the Supplementary Material (Section 8, p. S10), the model and the solution are described in detail with all assumptions outlined. In brief, the two-compartment model has two governing equations:

$$V_{i}\frac{dC_{c}(t)}{dt}=\mathrm{CBF}(t)[C_{a}(t)-C_{v}(t)]-{\mathrm{PS}}_{c}[C_{i}(t)-C_{e}(t)],$$ (1)

$$V_{e}\frac{dC_{e}(t)}{dt}={\mathrm{PS}}_{c}[C_{i}(t)-C_{e}(t)]-{\mathrm{CMRO}}_{2}(t).$$ (2)

Here $C_{i}$ and $C_{e}$ refer to volume-averaged intravascular (blood plasma) and extravascular unbound molecular oxygen ($O_{2}$) concentrations, respectively; $C_{a}$, $C_{v}$, and $C_{c}$ refer to arterial, venous and capillary $O_{2}$ concentrations, respectively, that include both $O_{2}$ bound to hemoglobin and unbound $O_{2}$ dissolved in blood plasma; $V_{i}$ and $V_{e}$ refer to the volumes of the intravascular and extravascular compartments within the entire tissue volume, respectively, with $V_{t}$ ($V_{t}=V_{i}+V_{e}$) and $V_{i}$ and $V_{e}$ usually defined per 100 g of tissue; ${\mathrm{PS}}_{c}$ is a mass transfer proportionality parameter that is often written as the product of capillary $O_{2}$ permeability, P (cm/s), and capillary surface area, $S_{c}$ [${\mathrm{cm}}^{2}/100\;g$ tissue]. In the physiological oxygen range, the concentrations $C_{e}$ and $C_{i}$ [Eq. (2)] are proportional to $p_{e}O_{2}$ and $p_{i}O_{2}$.

Equation (1) relates the rate of change in the capillary oxygen ($C_{c}$) to the oxygen influx due to CBF and oxygen loss via diffusion into the extravascular space. Equation (2) is a mass-balance statement that accounts for oxygen diffusion from capillaries and oxygen consumption in the extravascular space. In this work, only Eq. (2) was used; Eq. (1) is shown as a reminder of the comparatively indirect way by which ${\mathrm{CMRO}}_{2}$ is deduced in the BOLD fMRI and fNIRS methods.

To determine ${\mathrm{CMRO}}_{2}$ as a function of time, Eq. (2) was evaluated numerically with experimental inputs of $C_{i}$ and $C_{e}$, which in turn were derived from phosphorescence lifetime measurements. The time-derivative $dC_{e}/dt$ was calculated using the finite difference method. The code was written in MATLAB. The values of the constants in the model, e.g., $V_{e}$ and ${\mathrm{PS}}_{c}$, were obtained using published microscopic physiological parameters and the Krogh-Erlang cylinder model^18^ (Supplementary Material, p. S16). The choice of ${\mathrm{PS}}_{c}$ and its effect on the magnitude and temporal profile of ${\mathrm{CMRO}}_{2}$ are discussed below.

## 3 Results and Discussion

### 3.1 Measurement Method

In this work, we developed a new variant of the phosphorescence quenching method^36^ for concurrent measurements of oxygen concentrations in the intravascular and extravascular compartments of rat brain. We employed two dendritic polyethyleneglycol-coated phosphorescent probes, Oxyphors,^30^^,^^31^ that do not cross the blood brain barrier^21^^,^^22^^,^^27^ and have minimally overlapping absorption and emission spectra. Thus, phosphorescence signals from the two probes can be retrieved with no cross-talk, and because one probe circulated in blood and the other resided in the extravascular space, $p_{i}O_{2}$ and $p_{e}O_{2}$ could be measured concurrently.

Excitation of a mixture of PtR4 and PtG4 at 635 nm results only in phosphorescence of PtG4 [Fig. 1(c)]. However, excitation at 517 nm, aimed at the absorption Q band of PtR4, induces a weak but nonnegligible emission from PtG4. Therefore, several optical filters were used to remove unwanted signals from each channel (Supplementary Material, p. S8). As a result, the phosphorescence spectrum of PtR4 was attenuated by nearly 60% [Fig. 1(c)]. This narrowing of the spectral range, coupled with a lower molar extinction coefficient and a lower emission quantum yield, caused the PtR4 signal to be 10–15 times weaker than that of PtG4, even when equal amounts of the probes were dissolved in an optically clear medium, such as an aqueous solution in optical cells. *In vivo* the difference between PtR4 and PtG4 was further exacerbated by stronger attenuation of excitation at 517 nm and of the phosphorescence of PtR4 due to endogenous tissue absorption. Thus, the phosphorescence signal of PtR4 was 10–20 times weaker than that of PtG4 even when PtR4 was injected in the brain in 10-fold higher concentration. Consequently, SNR in Ch2 [Fig. 1(c)] was significantly lower than that in Ch1, irrespective of whether PtR4 was injected into blood or interstitial space. Despite this challenge, ${\mathrm{pO}}_{2}$ readings were obtained at a rate of 7 Hz for two channels and were sufficient for resolving transients in respiratory and vascular activity upon neuronal activation. In most experiments, PtR4 was delivered intravascularly, and its concentration could be increased via additional injections during the experiments through the catheter, if necessary.

### 3.2 Recordings of p_e_O_2_, p_i_O_2_, and CBF

**Fig. 2:**
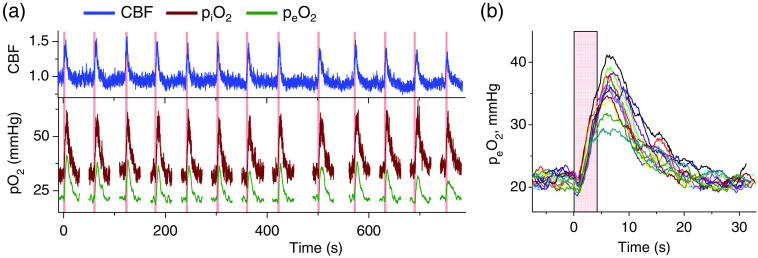
Raw unprocessed data obtained in an experiment consisting of 13 consecutive forepaw stimulations, accompanied by panel (a) CBF normalized to the initial baseline, and $p_{i}O_{2}$ and $p_{e}O_{2}$ measurements in the somatosensory area of the rat brain. (b) Overlapping traces of $p_{e}O_{2}$, synchronized at the start of the stimulation (t=0). The stimulation period is shown by a pink rectangle.

An example of raw unprocessed traces of $p_{e}O_{2}$ (Ch1/PtG4), $p_{i}O_{2}$ (Ch2/PtR4), and CBF during a series of stimulations is shown in Fig. 2(a). Importantly, the ABP was not affected by stimulations (see Supplementary Material, p. S23). The recording of ${\mathrm{pO}}_{2}$ was paused between the stimulations to minimize brain exposure to the excitation light and associated phototoxicity. All three parameters exhibited a sharp rise after stimulation, followed by a decrease to the baseline level. Immediately upon the start of the stimulation, $p_{e}O_{2}$ exhibits a characteristic “initial dip” due to the rapid increase in the local oxygen consumption by activated cells [Fig. 2(b)]. On average, the dip minima occurred 0.8±0.1  s after the onset of stimulation, in agreement with previous measurements.^22^^,^^23^ The observation of such dips in microscopic experiments typically requires the averaging of hundreds of simulation trials;^22^^,^^23^ in our experiments, remarkably, the dips could be seen in practically all individual stimulation events. These signals were strong in part because they originated from a large excitation volume that likely encompassed the entire activation region. However, this volume also includes cells with oxygen consumption that does not change significantly upon activation, and hence, the amplitudes of the dips are expected to be lower due to “signal dilution.”

The average baseline $p_{i}O_{2}$ was 33.5±0.5  mmHg (mean for 13 events, measured during the 4s prior to stimulation, and ± standard deviation of the mean). This baseline $p_{i}O_{2}$ is on the lower side of $p_{i}O_{2}$ values reported previously,^21^^–^^23^^,^^37^ possibly due to the use of α-chloralose anesthetic. Anesthesia is known to have pronounced effects on tissue ${\mathrm{pO}}_{2}$.^37^^,^^38^ The average baseline $p_{e}O_{2}$ was 21.3±0.2  mmHg, and the resulting $p_{i}O_{2}/p_{e}O_{2}$ difference was 12.2±0.4  mmHg. In previous studies performed on rat brain and utilizing 2PLM and isoflurane as the anesthetic, ${\mathrm{pO}}_{2}$ drops of ∼15  mmHg were seen over 25-μm distances from isolated arterioles, corresponding to the gradients of ∼0.6  mmHg/μm.^24^ Measurements around capillaries in rat brain using microelectrodes (with pentobarbital anesthesia) produced somewhat lower gradients, ∼0.45  mmHg/μm.^19^ In the present case, $p_{i}O_{2}$ values correspond mainly to the capillary ${\mathrm{pO}}_{2}$ because the total volume of blood in capillaries is larger, and optical excitation in thin capillaries is much more efficient than in large vessels. The $p_{e}O_{2}$ represents the entire extravascular space within the excitation volume. Assuming an average intercapillary distance of 60  μm,^39^ the measured mean $p_{i}O_{2}$ and $p_{e}O_{2}$ values result in the gradient of ∼0.8  mmHg/μm and consequently correspond to a relatively high baseline ${\mathrm{CMRO}}_{2}$. This larger apparent gradient is consistent with sampling at a greater depth in the cortex and therefore higher neuron density and higher rates of oxygen consumption. Note, however, that we cannot unambiguously exclude contributions from systematic errors (see Sec. 3.5) in the measurements of $p_{i}O_{2}$ and/or $p_{e}O_{2}$, which will be further analyzed and corrected as the method develops.

In a number of cases, only four to five consecutive stimulations were successful, after which the brain lost responsiveness. The latter observation suggests that measurements could have been causing some tissue damage. However, our attempts to correlate the early loss of response with the duration of photo-exposure and/or probe dose were not conclusive. On balance, the loss of responsiveness appeared to be more frequent in animals for which green light excitation (517 nm and Ch2) was used in combination with PtR4 delivered into the extravascular space.

### 3.3 Calculation of CMRO_2_

According to Eq. (2), ${\mathrm{CMRO}}_{2}$ can be derived from the experimentally measured values of $C_{e}$ and $C_{i}$ and the time derivative, $dC_{e}/dt$, for extravascular oxygen. In previous work, the evaluation of this time derivative, as well as of the derivative $dC_{c}/dt$ in Eq. (1), was not possible. Therefore, the derivatives were omitted, and ${\mathrm{CMRO}}_{2}$ was computed from instantaneous values of CBF, $C_{a}$ and $C_{v}$ (e.g., ${\mathrm{CMRO}}_{2\;}=\mathrm{CBF}\times [C_{a}-C_{v}]$). This approach effectively assumes that the system is (always) in a steady state, even during functional activation. As a result, the deduced dynamics of ${\mathrm{CMRO}}_{2}$ are inherently tied to that of CBF.^12^^–^^14^^,^^40^

**Fig. 3:**
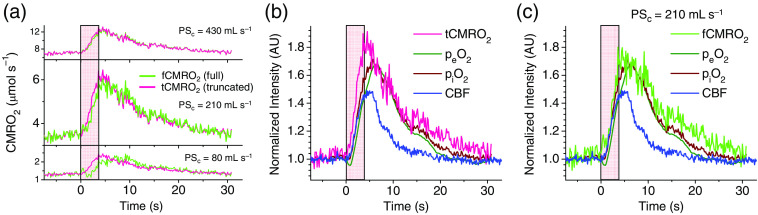
Averaged traces obtained in the experiment shown in Fig. 2. The stimulation period is shown by the pink rectangle. All traces are synchronized at the start of the stimulation (t=0). (a) Computed traces of ${\mathrm{fCMRO}}_{2}$ and ${\mathrm{tCMRO}}_{2}$ (see the text for definitions) for three different values of parameter ${\mathrm{PS}}_{c}$. Traces of CBF, $p_{e}O_{2}$, $p_{i}O_{2}$, ${\mathrm{tCMRO}}_{2}$ (b), and ${\mathrm{fCMRO}}_{2}$ (c) (calculated for ${\mathrm{PS}}_{c}=210\;\mathrm{mL}/s$) normalized by their baseline values to emphasize relative changes in the magnitudes.

**Fig. 4:**
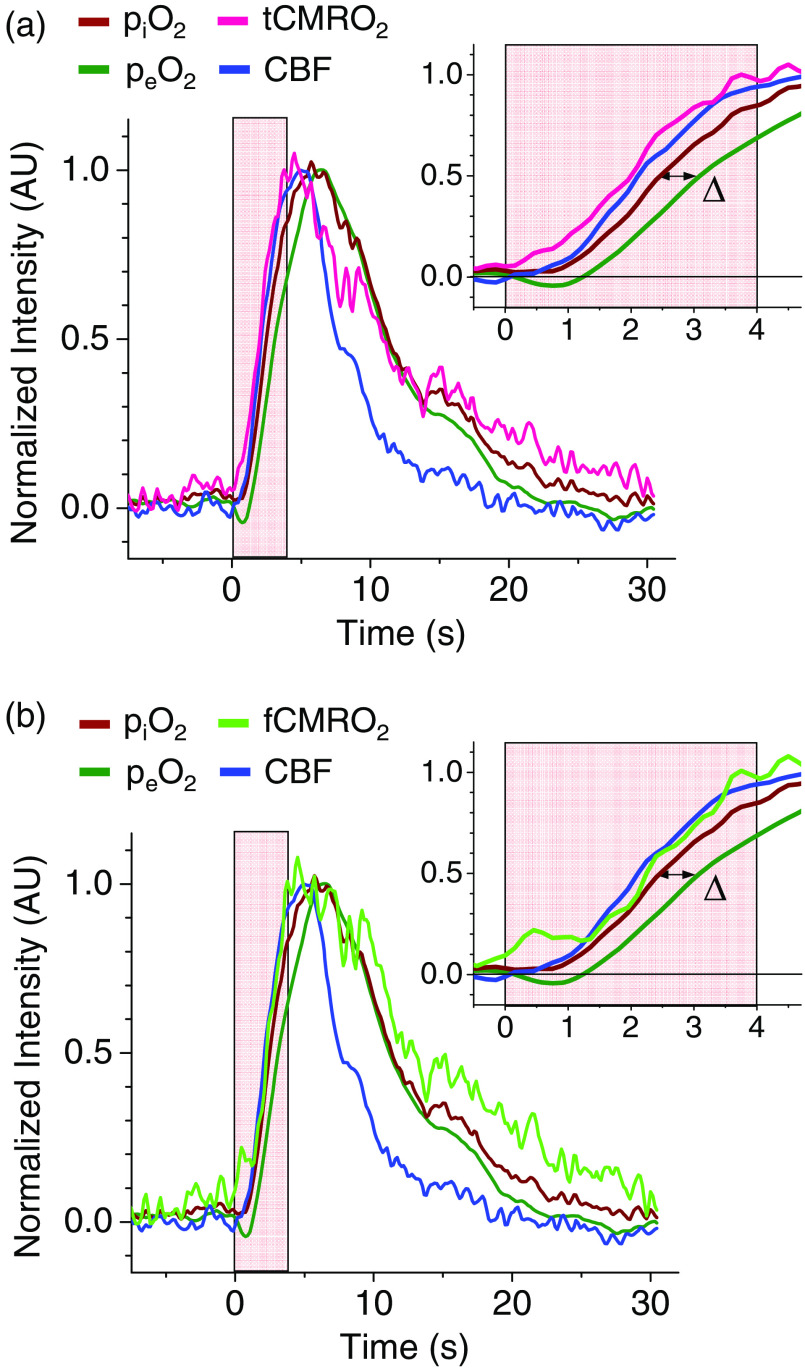
Normalized traces of $p_{i}O_{2}$, $p_{e}O_{2}$, CBF, and ${\mathrm{CMRO}}_{2}$. Time courses of (a) ${\mathrm{tCMRO}}_{2}$ and (b) ${\mathrm{fCMRO}}_{2}$ (computed for ${\mathrm{PS}}_{c\;}=210\;\mathrm{mL}/s$) relative to the time courses of $p_{i}O_{2}$, $p_{e}O_{2}$, and CBF. The stimulation period is shown by a pink rectangle. All traces were smoothed and synchronized at the start of the stimulation (t=0) and normalized by their maximal amplitudes for timing analysis (Supplementary Material, p. S20). Insets: initial period after activation. Δ: delay (at half-maximum) between the traces of $p_{i}O_{2}$ and $p_{e}O_{2}$.

The ability to independently measure $C_{e}$ and its time derivative ($dC_{e}/dt$) is a new feature that makes our technique unique and potentially more accurate than methods used previously. To distinguish between the traditional and present approaches, we introduce the terms full- and truncated-dynamic ${\mathrm{CMRO}}_{2}$ (${\mathrm{fCMRO}}_{2}$ and ${\mathrm{tCMRO}}_{2}$). ${\mathrm{fCMRO}}_{2}$ is computed using the complete Eq. (2), including the derivative $dC_{e}/dt$; ${\mathrm{tCMRO}}_{2}$ is computed ignoring the time derivative. With the same input data for $C_{e}$ and $C_{i}$, the difference between the computed ${\mathrm{fCMRO}}_{2}$ and ${\mathrm{tCMRO}}_{2}$ depends on coefficients $V_{e}$ and ${\mathrm{PS}}_{c}$ [Eq. (2)]. The value of $V_{e}$ (tissue extravascular volume) for brain tissue is well constrained^41^^,^^42^ (Supplementary Material, p. S16). By contrast, the published values for the product ${\mathrm{PS}}_{c}$ vary widely, reflecting the fact that the assessment of the oxygen diffusion coefficient in tissue is notoriously difficult. Unfortunately, both the magnitude and temporal profile of ${\mathrm{CMRO}}_{2}$ are strongly affected by the choice of ${\mathrm{PS}}_{c}$. Example traces of ${\mathrm{fCMRO}}_{2}$ and ${\mathrm{tCMRO}}_{2}$ based on the same input data, but using different values of ${\mathrm{PS}}_{c}$, are shown in Fig. 3(a). These profiles and all other traces shown in Figs. 3 and 4 were computed by averaging the ${\mathrm{CMRO}}_{2}$ traces for 13 stimulations (Fig. 2), whereby the individual ${\mathrm{CMRO}}_{2}$ traces were obtained by solving Eq. (2) using the respective traces of $p_{i}O_{2}$ and $p_{e}O_{2}$. The solutions were obtained with or without inclusion of the time derivative.

When ${\mathrm{PS}}_{c}$ is large, the first term on the left side of Eq. (2) is dominant, and the profiles of ${\mathrm{fCMRO}}_{2}$ and ${\mathrm{tCMRO}}_{2}$ strongly overlap [Fig. 3(a)]. However, for smaller ${\mathrm{PS}}_{c}$, ${\mathrm{fCMRO}}_{2}$ increasingly lags behind ${\mathrm{tCMRO}}_{2}$, as the contribution of the term $V_{e}\times dC_{e}/dt$ becomes nonnegligible. This time-lag effect is a direct consequence of the inclusion of the time-derivative $dC_{e}/dt$. The solution of Eq. (2) contains an exponential term with time constant $\tau =V_{e}/{\mathrm{PS}}_{c}$ that is an intrinsic feature of the perturbation response kinetics. For example, if the system starts in a steady state ($dC_{e}/dt=0$) and then $p_{i}O_{2}$ increases (or decreases) instantly in stepwise fashion, then a new steady-state ${\mathrm{CMRO}}_{2}$ is reached in exponential fashion with time constant τ. Notably, the delay between the rise of $p_{i}O_{2}$ versus $p_{e}O_{2}$ depends on the parameter ${\mathrm{PS}}_{c}$ and on the temporal variation of physiological responses. In addition, the parameter ${\mathrm{PS}}_{c}$ affects the absolute magnitude of ${\mathrm{CMRO}}_{2}$. In our analysis, we used the value ${\mathrm{PS}}_{c}=210\;\mathrm{mL}/s/100\;g$ tissue. This choice and the choices of other values were made based on the published microscopic physiological parameters and the Krogh-Erlang cylinder model (Supplementary Material, p. S16).

### 3.4 Magnitude and Dynamics of Responses

In contrast to prior work, our experiments enable the calculation of ${\mathrm{CMRO}}_{2}$ solely from oximetry data without reliance on CBF. Additionally, we independently measured CBF using LSCI. Thus, taken together, our measurements provide a unique opportunity to compare the response of all four parameters, CBF, $p_{i}O_{2}$, $p_{e}O_{2}$, and ${\mathrm{CMRO}}_{2}$, and thereby uncover physiologically important information about the response magnitude and dynamics that is not obtainable by other methods.

${\mathrm{fCMRO}}_{2}$ and ${\mathrm{tCMRO}}_{2}$ were computed using the $p_{i}O_{2}$ and $p_{e}O_{2}$ data from 13 stimulations (Fig. 2). The CBF, $p_{i}O_{2}$, $p_{e}O_{2}$, and ${\mathrm{CMRO}}_{2}$ traces were averaged and normalized either by the respective baseline values [Figs. 3(b) and 3(c)] or by the maximal amplitudes, i.e., the difference between the baseline and the peak value of the trace (Fig. 4). Baseline normalization helps to visualize relative changes in magnitude, whereas normalization by the amplitude has been used previously for timing analysis.^43^ Both ${\mathrm{fCMRO}}_{2}$ and ${\mathrm{tCMRO}}_{2}$ were computed to illustrate the difference between the two methods of calculation, particularly in relation to the timing analysis.

Prior to activation, CBF, $p_{i}O_{2}$ and $p_{e}O_{2}$ and ${\mathrm{CMRO}}_{2}$ are at their baseline levels; the baseline ${\mathrm{CMRO}}_{2}$ value is ∼3.5  μmol/s/100  g tissue [Fig. 3(a)], which is within the range of reported baseline ${\mathrm{CMRO}}_{2}$ values for the rat brain.^44^^,^^45^ Activation causes an immediate increase in the energy consumption by neurons and other cells, and the mitochondrial oxidative phosphorylation responds nearly instantaneously by elevating the rate of adenosine triphosphate (ATP) synthesis and, consequently, ${\mathrm{CMRO}}_{2}$. This rise in the energy consumption is the key trigger that sets in motion the cascade of events that define the time courses of all measured parameters.

With respect to the timing analysis, the ${\mathrm{tCMRO}}_{2}$ and ${\mathrm{fCMRO}}_{2}$ models will exhibit the same behavior in the limit of large ${\mathrm{PS}}_{c}$. This behavior is also the same as for the traditional steady-state model because the time-derivative term in Eq. (2) vanishes in the large ${\mathrm{PS}}_{c}$ limit. However, it should be noted that the absolute magnitude of ${\mathrm{CMRO}}_{2}$ also depends on ${\mathrm{PS}}_{c}$. Ultimately, it is desirable that the chosen value of ${\mathrm{PS}}_{c}$ should give the correct ${\mathrm{CMRO}}_{2}$ dynamics and magnitude.

First, it is useful to examine the timing of the events according to the ${\mathrm{tCMRO}}_{2}$ model, which implies that the rate of change in CBF, $p_{i}O_{2}$, and $p_{e}O_{2}$ is much lower than the rate of oxygen diffusion over the distance separating the source (capillaries) and the consumption sites (mitochondria in cells). The normalized traces of all measured parameters and ${\mathrm{tCMRO}}_{2}$ are shown in Fig. 4(a).

The rapid rise in ${\mathrm{CMRO}}_{2}$ due to functional stimulation results in local depletion of oxygen, which is manifested by the “initial dip” in the trace of $p_{e}O_{2}$ with a minimum that is reached at ∼1  s from the start of the activation. Similar delays between the stimulation onset and the dip have been reported previously.^22^^,^^23^ The increase in the energy consumption results in the vascular response of up-stream arterioles, inducing their dilation, via a pathway known as neurovascular coupling.^2^^,^^17^ As a result, local CBF begins to rise, bringing new oxygenated blood to the activation region. The propagation of the neurovascular coupling cascade is much faster than the time resolution of our measurements, while our experimental traces clearly show, in spite of the noise, that the rise in CBF is delayed relative to the rise in ${\mathrm{CMRO}}_{2}$ [Fig. 4(a)]. Presumably, this delay is associated with the time needed for arterioles to dilate and for the blood to reach the activation region.

The rise in $p_{i}O_{2}$ begins nearly simultaneously with the rise in CBF, reflecting the fact that arterial blood flowing into the region contains more oxygen. The incoming oxygen diffuses from the capillaries into the extravascular space, and its flux compensates for and then exceeds the rate of oxygen consumption, causing $p_{e}O_{2}$ to curve around the minimum (dip region) and start rising. The subsequent trace of $p_{e}O_{2}$ follows that of $p_{i}O_{2}$ but with a delay Δ∼0.6  s (measured at half-maximum), consistent with previous observations.^43^ Delay Δ depends on the interplay of dynamics between CBF and ${\mathrm{CMRO}}_{2}$ and the diffusion of oxygen from the blood to the consumption sites. As noted above, in the limit of large ${\mathrm{PS}}_{c}$, the diffusion is fast, and delay Δ is set almost solely by the relative changes in CBF versus ${\mathrm{CMRO}}_{2}$.

Soon after the end of the stimulation, all four parameters reach their peak values and begin to decline. At its maximum, ${\mathrm{CMRO}}_{2}$ is ∼6.0  μmol/sec/100  g tissue [Fig. 3(a), middle panel], i.e., ∼1.8 times higher than its baseline value [Fig. 3(b)], and the respective increase in CBF is ∼1.5×. In other experiments (n=3, averaged over 4 to 5 stimulations), the respective increases in CBF and ${\mathrm{CMRO}}_{2}$ were reversed, e.g., ∼1.4× and ∼1.2×, respectively. The observation of a larger increase in ${\mathrm{CMRO}}_{2}$ compared with that in CBF is somewhat unusual, because many (but not all) previous studies have found that CBF response exceeds ${\mathrm{CMRO}}_{2}$ response in magnitude.^13^^,^^14^^,^^40^ It is possible that the inversion seen in our experiments was influenced by some systematic measurement errors (see Sec. 3.5). However, in principle, the inversion is not impossible because the amount of oxygen delivered to the activation area, even upon moderate increase in CBF, can still overwhelm the demand caused by a large increase in ${\mathrm{CMRO}}_{2}$ (due to the very high oxygen-carrying capacity of hemoglobin). Indeed, the ${\mathrm{pO}}_{2}$ traces showed characteristic “overshoots” similar in magnitude to overshoots reported previously.^23^ The overshoots have been hypothesized to be a consequence of the anatomical structure of the brain, whereby arterioles triggered by neurovascular coupling supply oxygen to a much larger volume than the actual activated region.^1^^,^^23^

After reaching their maxima, all of the parameters gradually return to the respective baselines. Interestingly, ${\mathrm{CMRO}}_{2}$, $p_{i}O_{2}$, and $p_{e}O_{2}$ decline at a similar rate, whereas the decrease in the CBF is somewhat steeper. This observation suggests that a signal for the cessation of arterial dilation is issued close to the time when ${\mathrm{CMRO}}_{2}$ levels off and begins to fall, but before it drops significantly. It appears that the system sends a feedback signal to attenuate arterial blood supply when oxygen in the pool is still sufficient for maintaining ${\mathrm{CMRO}}_{2}$ at an elevated level. In this regard, it is important to stress that ${\mathrm{CMRO}}_{2}$ is independent of ${\mathrm{pO}}_{2}$ over the physiological range.^46^^,^^47^ Changes in CBF and/or ${\mathrm{pO}}_{2}$ neither affect nor control ${\mathrm{CMRO}}_{2}$. Rather, it is ${\mathrm{CMRO}}_{2},$ which is set by the rate of ATP consumption through changes in the cellular energy state,^48^ that drives the events in the context of our experiment. The vascular system merely responds to changes in ${\mathrm{CMRO}}_{2}$, which depends on changes in the energy state to keep the oxygen supply above a certain threshold level.

The full-dynamic model suggests a more complex profile of ${\mathrm{CMRO}}_{2}$, particularly near the onset of the stimulation [Fig. 4(b)]. In this case, a clear initial rise in ${\mathrm{fCMRO}}_{2}$ triggers an increase in CBF. This initial rise in ${\mathrm{fCMRO}}_{2}$ is followed by a flattening of the response for a brief period (0.3 to 1.3 s) and finally by a steady rise. As in the case of ${\mathrm{tCMRO}}_{2}$ [Fig. 4(a)], the initial changes in ${\mathrm{fCMRO}}_{2}$ trigger an increase in CBF; however, starting from ∼1.5  s ${\mathrm{fCMRO}}_{2}$ does not lead to CBF, but instead their traces roughly overlap. The dynamics on longer time scales discussed above are essentially the same for both models.

The peak ${\mathrm{fCMRO}}_{2}$ value is somewhat lower than that predicted by the truncated model, showing an ∼1.7× increase relative to the baseline level [Fig. 3(c)]. The subsequent decline in ${\mathrm{CMRO}}_{2}$ is also slightly different, i.e., the trace in Fig. 4(b) versus Fig. 4(a) is more delayed in time. However, these differences are not large, and the poststimulation profiles varied significantly between animals, making comparisons difficult at this stage.

The differences between the traces of ${\mathrm{tCMRO}}_{2}$ and ${\mathrm{fCMRO}}_{2}$ offer new means for exploring underlying physiological assumptions in the models. While recognizing the limited time resolution of our measurements and uncertainty due to the noise, the temporal behavior of ${\mathrm{fCMRO}}_{2}$ clearly does not conform to traditional expectations. It is tempting to speculate about the origin of the initial feature (“bump”) in the profile of the dynamic ${\mathrm{CMRO}}_{2}$. One possibility is that the choice of ${\mathrm{PS}}_{c}$ (210  mL/s) in our estimations was not correct, and with larger ${\mathrm{PS}}_{c}$ values, the full-dynamic model would converge to the traditional steady-state limit. However, the baseline ${\mathrm{CMRO}}_{2}$ magnitude would then be larger, whereas essentially all prior estimates of ${\mathrm{PS}}_{c}$ give values in the range of 35 to 230  mL/s.^49^ It is also possible that systematic measurement errors could lead to overestimation of oxygen gradients. If the actual gradients are lower, then the same baseline ${\mathrm{CMRO}}_{2}s$ would correspond to higher ${\mathrm{PS}}_{c}s$. At this stage, we do not advocate for any particular set of assumptions. Rather, we emphasize that evidence from our qualitatively new methodology takes first steps toward a more critical examination of models/model inputs.

### 3.5 Limitations

As mentioned throughout the text, several limitations in the current implementation of the method could cause systematic measurement errors.

1. First and foremost, the optical frequencies (wavelengths) used for probing $p_{i}O_{2}$ and $p_{e}O_{2}$ in our experiment were different (by design) to eliminate the cross-talk between the two measurement channels. However, the effective depth of sampling by light is wavelength-dependent due to the endogenous absorption and scattering. Strong optical heterogeneity of tissue makes it difficult to evaluate the difference in the sampling depth, but estimates based on brain optical properties suggest that the sampling depth for PtR4 was ∼3× less than for PtG4. Consequently, the two probed volumes were unequal, which could affect quantification of the oxygen gradients.
  In the future, it should be possible to select two probes excitable at the same wavelength, but having minimally overlapping phosphorescence spectra, both in the near-infrared region, wherein the endogenous optical absorption spectrum is nearly flat. Using appropriate optical filtering, the signals of the probes will be sampled independently, and the difference between the probed volumes will be minimized. A selection of dendritic oxygen probes with different optical parameters is available^30^^,^^31^^,^^50^ and will be tested in the future.
2. Because the brain tissue after the craniotomy was exposed to room-temperature air, a temperature gradient from the surface down was established. Hemoglobin affinity for oxygen, phosphorescence decay times, and oxygen quenching parameters of the probes are all temperature-dependent. Thus, the combination of the temperature gradient, which was unknown, with the different sampling depths for the two probes (see above) could have an effect on the measured apparent oxygen gradients. Again, minimizing the difference between the sampling volumes should decrease the associated systematic error.
3. One of the principal strengths of all-optical methodology is its minimal invasiveness. However, in our experiments, one of the probes had to be delivered by direct injection into the brain tissue close to the measurement site, potentially damaging the brain. To circumvent this problem in the future, it should be possible to introduce the extravascular ${\mathrm{pO}}_{2}$ probe by injection into the cisterna magna,^50^ far away from the measurement site, and thus avoid tissue damage.

## 4 Conclusions

We presented the first demonstration of real-time tracking of tissue oxygen gradients concurrently with local blood flow during and after functional activation of rat brain. In contrast to measuring the average oxygen concentration, the technique provides a direct observation window on the rate of the brain metabolism, which is assessed independently from CBF. The method utilizes a combination of the phosphorescence quenching oximetry, employing two optically distinguishable membrane-impermeable oxygen probes, with LSCI. In spite of technical challenges, the technique proved capable of extracting information about the timing of metabolic events accompanying neuronal activation, not obtainable by any other existing method. The innovative method also permitted direct comparison of full-dynamic versus truncated-dynamic (steady state) models based on the same experimental input. As technology develops, we anticipate that the method will become more broadly available for testing drugs and other effectors of brain metabolism, and it should permit more rigorous examination of metabolism models.
