# Supplementary material for: Real-time tracking of brain oxygen gradients and blood flow during functional activation
Source: Neurophotonics. 2022 Nov 28;9(4):045006. doi: 10.1117/1.NPh.9.4.045006 (PMC9704417; doi:10.1117/1.NPh.9.4.045006)

SUPPLEMENTARY MATERIAL

Real-time Tracking of Brain Oxygen Gradients and Blood Flow during Functional Activation

Sang Hoon Chong,[1] Yi Hong Ong,[1,2] Mirna El Khatib,[3,4] Srinivasa Rao Allu,[3,4] Ashwin B. Parthasarathy,[5] Joel H. Greenberg,[6] Arjun G. Yodh,*[1] and Sergei A. Vinogradov*[3,4]

[1] *Department of Physics and Astronomy, University of Pennsylvania, Philadelphia, PA 19104, USA*

[2] *Department of Radiation Oncology, University of Pennsylvania, Philadelphia, PA 19104, USA*

[3] *Department of Biochemistry and Biophysics, Perelman School of Medicine, University of Pennsylvania, Philadelphia, PA 19104, USA*

[4] *Department of Chemistry, School of Arts and Sciences, University of Pennsylvania, Philadelphia, PA 19104, USA*

[5] *Department of Electrical Engineering, University of South Florida, Tampa, FL 33620, USA*

[6] *Department of Neurology, University of Pennsylvania, Philadelphia, PA 19104, USA*

E-mail: vinograd.upenn@gmail.com, yodh@physics.upenn.edu

Table of Contents

1. General information ________________ S3
2. Synthesis of Oxyphor PtR4 ________________ S3
3. Photophysical properties of Oxyphor PtR4 ________________ S6
4. Oxygen quenching properties of Oxyphor PtR4 ________________ S8
5. Two-channel phosphorometer ________________ S8
6. Laser speckle imaging optics ________________ S9
7. Stimulus protocol ________________ S9
8. Modeling of oxygen diffusion and calculation of $CMRO_2$ ________________ S10
9. Selection of PS_c ________________ S16
10. Timing analysis ________________ S20
11. Records of CBF and ABP during stimulation ________________ S23
12. Potential measurement artifacts: oxygen consumption, probe distribution ________________ S24
13. NMR spectra ________________ S25

1. General information

All solvents and reagents were purchased from standard commercial sources and used as received. Polyethyleneglycol-amine monomethyl ether (MeO-PEG-NH_2), Av. MW 1000, was obtained from Laysan Bio. CBzNH-AG^{1}OH and PtTPP-OH were synthesized as described previously.[30, 31] Size-exclusion chromatography (SEC) was performed using Bio-Beads S-X1 (40-80 μm bead size, Biorad) using THF as a mobile phase. Column chromatography was performed using Selecto™ silica gel (Fisher Scientific). Analytical thin-layer chromatography (TLC) was carried out using silica gel matrix with a fluorescent indicator (layer thickness 200 μm) on aluminum support (SiliCycle). ^{1}H and ^{13}C NMR spectra were recorded on Bruker Neo400 (400.17 MHz for ^{1}H, 100.6 MHz for ^{13}C), UNI600 (600.1 MHz for ^{1}H, 150.9 MHz for ^{13}C) and UNI500 (500.4 MHz for ^{1}H, 125.8 MHz for ^{13}C) spectrometers. Deuterated solvents ($CDCl_3$, CD_2Cl_2, DMSO-d_6) were purchased from Cambridge Isotope Laboratories, Inc. The NMR data were analyzed using MestReNova software (Mestrelab Research). Mass spectra were recorded on a MALDI-TOF Bruker Daltonics Microflex LRF instrument using α-cyano-4-hydroxycinnamicacid (CCA) as a matrix (positive-ion mode). Samples were prepared by mixing a solution of the analyte in THF or MeOH (10 μL, ~1 mM) with a solution of the matrix (100 μL, 10 mg/ml, 0.053 M) in CH_2Cl_2/2-propanol (9:1). The sample, approximately 1 μL, was deposited on the probe tip, dried and analyzed.

Artificial cerebrospinal fluid (aCSF) was supplied by the Cerebrovascular Group in the Department of Anesthesiology and Critical Care at the University of Pennsylvania. The ingredients for preparation of 1L of aCSF are listed below: diH2O (1L), KCl (0.220 g), $MgCl_2$ (0.132 g), $CaCl_2$ (0.221 g), urea (0.402 g), dextrose (0.665 g), NaCl (7.710 g), $NaHCO_3$ (0.207 g / 100 mL; added on the day of use).

2. Synthesis of Oxyphor PtR4

CBzNH-AG^1(GluO^{t}Bu)$_2$. To a solution of *N*-(benzyloxycarbonyl)-3,5-dicarboxyphenyl glycineamide, CBzNH-AG^{1}OH (0.744 g, 2 mmol), in DMF (20 ml), a solution of CDMT (0.878 g, 5.0 mmol) in DMF (4 ml) was added at 0°C, followed by immediate addition of N-methylmorpholine (1.1 mL, 10 mmol). The reaction mixture was stirred at 0°C for 1h, then a solution of glutamic acid di-*tert*-butyl ester hydrochloride (1.302 g, 4.4 mmol) in DMF (20 ml) was added, and the reaction mixture was stirred at room temperature for 48h. The solvent was removed in vacuum, the resulting oil was dissolved in CH_2Cl_2 (300 ml), washed with HCl aq. (1N, 100 ml), then with $NaHCO_3$ aq. (10%, 2×100 ml) and dried over Na_2SO_4. The product was purified by column chromatography (silica gel, CH_2Cl_2/MeOH, 25:1) to give the

title compound as a yellowish solid. Yield 1.22 g (78%). ^{1}H NMR (DMSO-d_6), δ (ppm): 1.40 (s, 18H), 1.43 (s, 18H), 1.89-2.10 (m, 4H), 2.35 (t, J = 7.6 Hz, 4H), 3.85 (d, J = 6.0 Hz, 2H), 4.33-4.39 (m, 2H), 5.07 (s, 2H), 7.23-7.39 (m, 5H), 7.61 (t, J = 6.4 Hz, 1H), 8.00 (s, 1H), 8.19 (s, 2H), 8.75(d, J = 7.2 Hz, 2H), 10.28 (s, 1H); ^{13}C NMR (DMSO-d_6), δ (ppm): 26.4, 28.1, 28.2, 31.8, 53.1, 66.0, 80.2, 81.1, 121.7, 128.2$_0$, 128.2$_5$, 128.8, 135.5, 137.5, 139.3, 157.1, 166.9, 168.8, 171.4, 171.9; MALDI-TOF (*m/z*): calcd. for $C_{44}H_{62}N_4O_{13}$: 854.4, found: 893.5 $[M+K]^+$.

Scheme S1. Synthesis of the Gen2 dendron **NH$_2$-AG^1(GluO^{t}Bu)$_2$**.

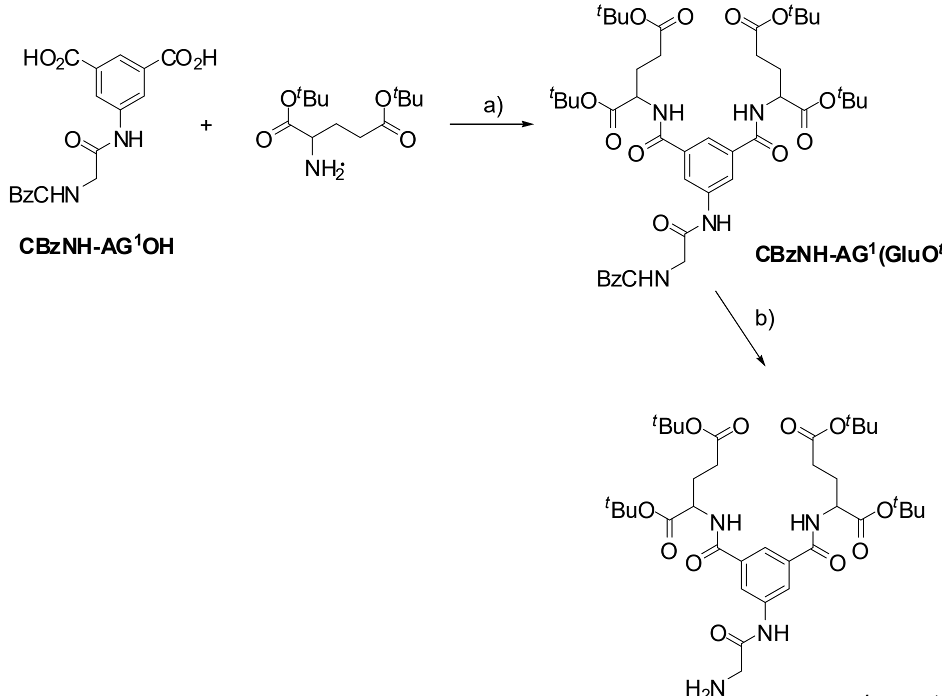

CBzNH-AG^{1}OH

CBzNH-AG^1(GluO^{t}Bu)$_2$

NH$_2$-AG^1(GluO^{t}Bu)$_2$

Reagents and conditions: (a) CDMT, *N*-methylmorpholine, DMF, r.t., 48 h (78%); (b) 10 wt. % palladium on carbon, THF, rt, 14-15 h (85%)

NH$_2$-AG^1(GluO^{t}Bu)$_2$. CBzNH-AG^1(GluO^{t}Bu)$_2$ (1.2 g, 1.40 mmol) was dissolved in THF:MeOH mixture (100 mL, 1:1), and the solution was deoxygenated by Ar bubbling during 10-15 min. Pd/C (10 wt%, 100 mg) was added to the solution, and the resulting mixture was flushed with hydrogen gas (H_2) and left under stirring in H_2 atmosphere (1 atm) for 14-15 h. The resulting mixture was passed through a Celite 545 column and the product was washed off the column with THF:MeOH mixture (200 mL, 1:1). Concentrating the washings afforded the title compound as a light-yellow solid. Yield: 0.86 g (85%). ^{1}H NMR (DMSO-d_6), δ (ppm): 1.38 (s, 18H), 1.41 (s, 18H), 1.88-1.96 (m, 2H), 1.99-2.07 (m, 2H), 2.33 (t, J

= 5.6 Hz, 4H), 3.29 (s, 2H), 4.31-4.35 (m, 2H), 7.99 (s, 1H), 8.23 (s, 2H), 8.72 (d, J = 6.0 Hz, 2H); ^{13}C NMR (DMSO-d_6), δ (ppm): 26.4, 28.1, 28.2, 31.8, 45.9, 53.1, 80.3, 81.2, 121.5, 121.6, 135.4, 139.2, 166.9, 171.4, 172.0, 172.8; MALDI-TOF (*m/z*): calcd. for $C_{36}H_{56}N_4O_{11}$: 720.4, found: 759.5 $[M+K]^+$.

Scheme S2. Synthesis of Oxyphor **PtR4**.

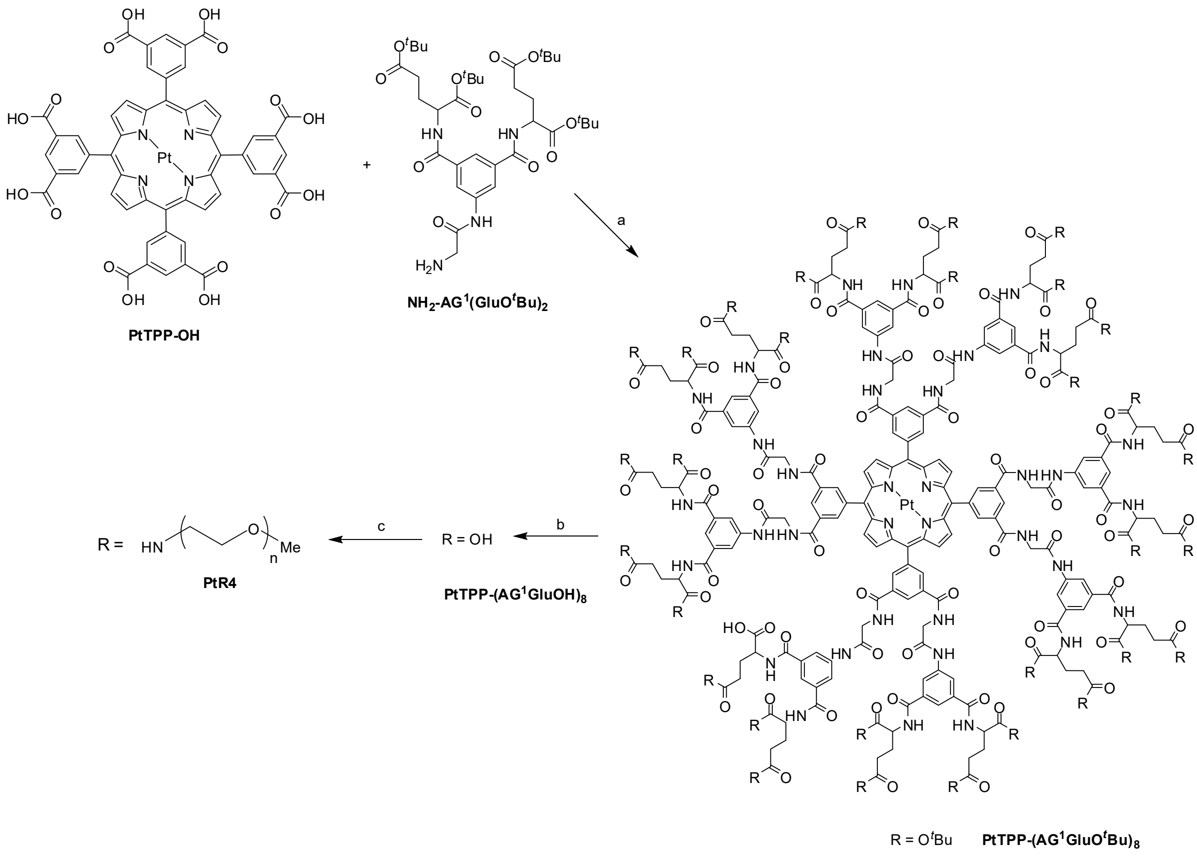

Reagents and conditions: (a) HBTU, DIPEA, DMF, r.t., 96 h (70%); (b) TFA, DCM, r.t., 2 h (90%); (c) NH_2-PEG (Av. MW~1000), HBTU, DIPEA, DMF, r.t., 10-12 days (63%).

PtTPP-(AG¹GluO^{t}Bu)$_8$. To a solution of PtTPP-OH (0.040 g, 0.034 mmol) in DMF (5 ml), HBTU (0.210 g, 0.554 mmol) was added, and the reaction mixture was stirred for 10 min at room temperature. *N,N*-diisopropylethylamine (0.19 mL, 0.554 mmol) was added to the mixture, immediately followed by addition of NH_2-AG^1(GluO^{t}Bu)$_2$ (0.4 g, 0.544 mmol). The resulting mixture was stirred at r.t. for 96 h. The reaction mixture was poured into aqueous HCl (5%, 30 ml). The resulting red precipitate was collected by centrifugation, washed with water (2×10 ml) and dried in vacuum. After purification by SEC (S-X1, THF),

the title porphyrin-dendrimer was isolated as a red solid. Yield: 0.162 g (70%). ^{1}H NMR (DMSO-d_6), δ (ppm): 1.34, 1.35 and 1.38 (s, 288H), 1.87-2.05 (m, 32H), 2.30-2.33 (m, 32H), 4.18 (br s, 16H), 4.31-4.35 (m, 16H), 6.62 (s, 4H), 6.86 (s, 8H), 7.99 (s, 8H), 8.21 (s, 16H), 8.71 (d, J = 6.0 Hz, 16H), 8.88-8.92 (m, 16H), 9.01 (s, 4H), 9.34 (s, 8H), 10.4 (s, 8H).

PtTPP-(AG^{1}GluOH)$_8$. To a solution of PtTPP-(AG^{1}GluO^{t}Bu)$_8$ (0.115 g, 0.017 mmol) in dichloromethane (10 ml), trifluoroacetic acid (5 mL) was added dropwise, and the mixture was stirred at r.t. for 2 h. The solvents were removed in vacuum, after which DCM (10 mL) was added and evaporated again. This procedure was repeated twice to remove the remaining traces of TFA, after which the resulting precipitate was dried. The product was used in the following step without further purification. Yield: 0.076 g (90%).

Oxyphor PtR4. To a solution of PtTPP-(AG^{1}GluOH)$_8$ (0.075 g, 0.015 mmol) in DMF (10 ml), HBTU (0.364 g, 0.96 mmol) was added, and the reaction mixture was stirred for 10 min at room temperature. DIPEA (0.67 mL, 3.84 mmol) was added to the mixture, followed by the addition of solid mPEG-Amine (MW 1000, 0.96 g, 0.96 mmol). The reaction mixture was stirred at room temperature for 7 days. Additional portions of HBTU (0.182 g, 0.48 mmol) and mPEG-Amine (MW 1000, 0.48 g, 0.48 mmol) were added to the mixture, and stirring continued for additional 5 days. The reaction mixture was poured into diethyl ether (100 mL) and sonicated. The obtained mixture was centrifuged, the supernatant was discarded, and the remaining dark red viscous material was dissolved in THF (10 mL) and subjected to SEC (Bioard S-X1, THF). The red-orange fraction was collected, the solvent was removed in vacuum and the product was dried. **PtR4** was isolated as a dark red solid. Yield: 0.345 g (63%).

3. Photophysical properties of Oxyphor PtR4

The absorption spectrum of PtR4 recorded in 50 mM phosphate buffer solution (pH 7.2) shows no traces of aggregation (Fig. S1). The photophysical properties of Oxyphor PtR4 are summarized in Table S1. The phosphorescence emission maximum is at 727 nm. The phosphorescence quantum yield was measured in aqueous solution (phosphate buffer, pH 7.2, 23°C) relative to the fluorescence of rhodamine 6G (Rh6G) in EtOH (ϕ_{fl} = 0.94) and found to be 0.053.

Table S1. Photophysical constants of Oxyphor PtR4.

Probe	Absorption, nm ($\varepsilon \times 10^{-4}$, $M^{-1}cm^{-1}$)[c]	Emission[a] λ_{max} nm	ϕ / τ_0 (μs)[b]
PtR4	403 (30.9)<br>512 (3.2)	660, 727	0.053 / 44

[a]The emission spectra were recorded in 50 mM phosphate buffer solution (pH 7.2, 23°C), deoxygenated by Ar. [b]The phosphorescence quantum yield (ϕ) was determined relative to the fluorescence of Rh6G (ϕ_{fl}=0.94) in EtOH; the phosphorescence lifetime at zero oxygen (τ_0) was measured using a solution deoxygenated by Ar. [c]The extinction coefficient was determined assuming the molecular weight of 36,410 Da.

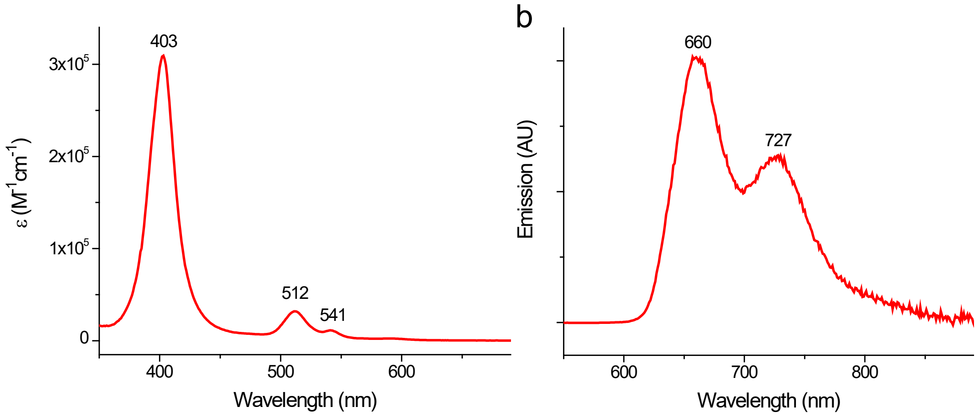

Figure S1. Absorption (a) and phosphorescence (b) spectra of Oxyphor PtR4 in 50 mM phosphate buffer solution (pH 7.1, λ_{ex}=512 nm).

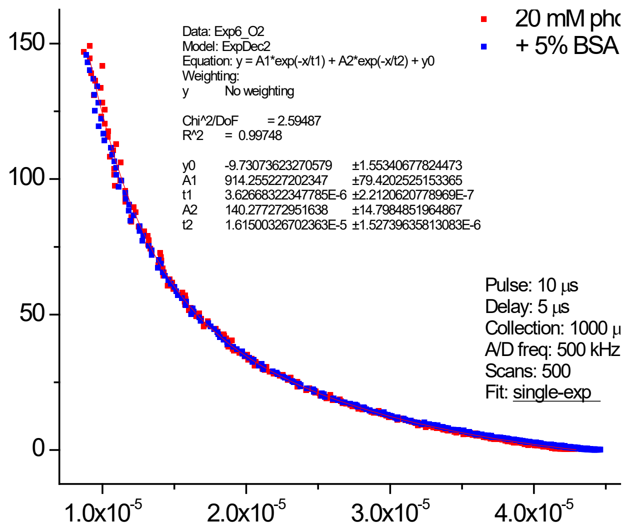

Figure S2. Phosphorescence quenching plot of Oxyphor PtR4.

4. Oxygen quenching properties of Oxyphor PtR4

The oxygen quenching plots of Oxyphor PtR4 were measured in buffered aqueous solutions (20 mM phosphate, pH 7.2) in the absence and presence of bovine serum albumin (BSA, 5%) and found to be nearly identical (Fig. S2). The data were fitted to an empirical bi-exponential form (shown in Fig. S2), and its coefficients were used to convert the phosphorescence lifetime measured *in vivo* to pO_2. The phosphorescence lifetime of Oxyphor PtR4 changes from ~8 μs at air saturation to ~43 μs at zero-oxygen at (36.8°C).

5. Two-channel phosphorometer

The instrument for two-color phosphorimetric measurements was constructed in house. The excitation sources were modulated laser diodes (Power Technology) operating at λ_{max}=630 nm (15 mW) and λ_{max}=517 nm (10 mW) for excitation of PtG4 and PtR4, respectively. Both diodes have the rise time of ~50 ns and can be controlled by standard TTL signals. The detectors were avalanche photodiodes (C12703-01, Hamamatsu; rise time 3.3 μs). The control of the data acquisition was performed using a digital-to-analog (DA)/analog-to-digital (AD) board (NI USB-6351, National Instruments; 1 MHz digitization frequency).

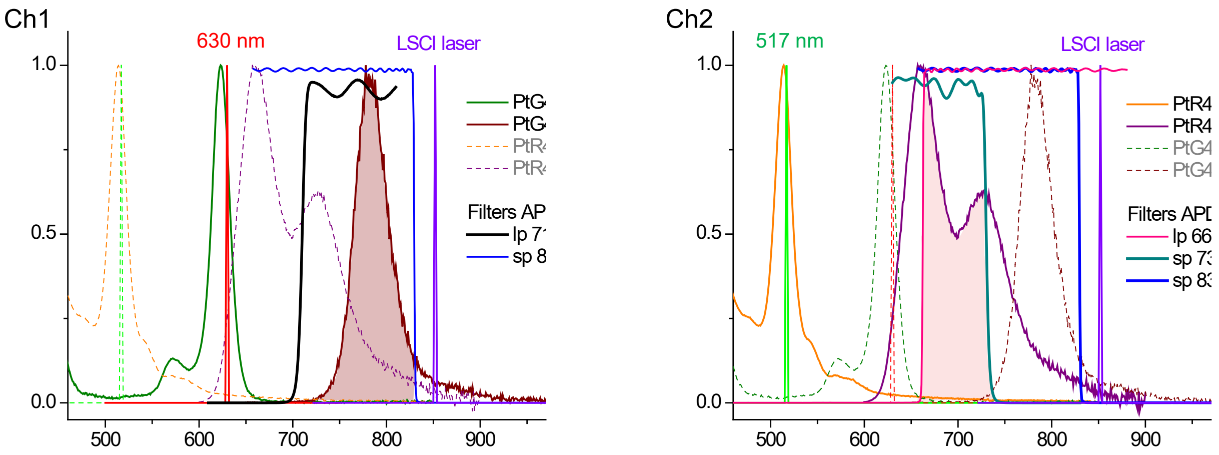

Figure S3. Optical absorption and emission spectra of the probes and associated laser lines and optical filters in Channels 1 and 2 of the phosphorometer. The spectral ranges seen by the detectors (APDs) are shown by shaded areas. 'lp' is the abbreviation for 'long-pass filter'.

The detectors, the digital board and the power supplies were located inside the instrument box, which was connected to a control computer by a USB cable. The front panel of the box contained two terminals for the cables conducting power and signals to the laser diodes and two optical ports for the optical fibers conducting the emitted light (phosphorescence) to the APDs, which were located inside the instrument box. The acquisition control and data analysis were performed by a program written in C/C++ (Qt, Nokia).

Each laser diode was placed inside a plastic holder tube, equipped with a lens (f=60 mm, ∅=12 mm), such that the beam could be focused into a spot (∅~0.2 mm) ~6 cm away from the tube's end. A single core plastic fiber (∅=4 mm, Fiberoptic Technology) ran on the side of the tube and held ~2 mm away from the excitation focus. The emitted light that entered the fiber was forwarded to an optical port in the instrument box. Inside the port the fiber tip was positioned next to a spherical lens (∅=10 mm) for light collimation, after which the light passed through a set of optical filters (∅=2.54 cm) and focused by another spherical lens (d=10 mm) on the entrance aperture of the APD (∅=3 mm). The optical filters configuration in the channels is shown in Fig. S3.

6. Laser speckle imaging optics

Laser speckle images were collected with an infinity-corrected optical system composed of two lenses with focal lengths of f=135 mm (Mitakon Zhongyi Mark II Lens for Nikon F, Zhong Yi Optics,

China) and f=75 mm (AC508-075-B, Thorlabs, Newton, NJ, USA). As result, the image was magnified by 1.8 times.

7. Stimulus protocol

Each stimulus cycle was 1 min-long and it consisted of 4s-long of baseline collection period, 4s-long electric forepaw stimulation period and 52s-long post-stimulus resting period (Fig. S4).

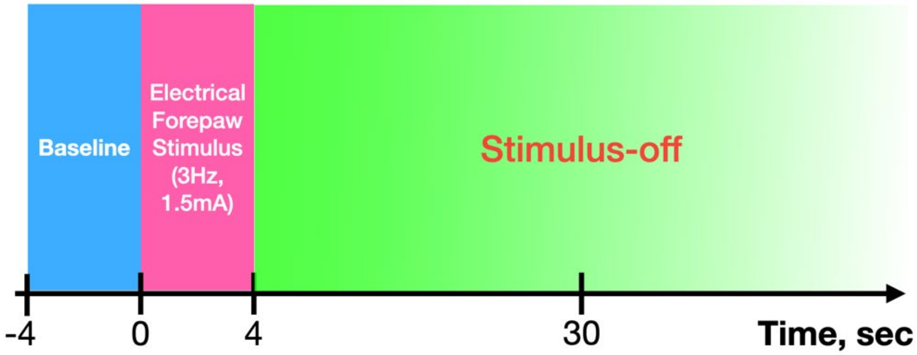

Figure S4. Timeline of the forepaw stimulation cycle.

8. Modeling of oxygen diffusion and calculation of $CMRO_2$

Oxygen transport equations. Modeling oxygen dynamics is an active area of research. For recent examples see ref. 49 as well as: (1) Lücker, A. et al. "The relative influence of hematocrit and red blood cell velocity on oxygen transport from capillaries to tissue." *Microcirculation* **24**(3), 2017: e12337. (2) Celaya-Alcala, J. T. et al. "Simulation of oxygen transport and estimation of tissue perfusion in extensive microvascular networks: Application to cerebral cortex." J. Cereb. Blood Flow & Metab. **41**(3), 2021: 656-669.

We adopted a simple approach whereby tissue is approximated by a cylindrical capillary tube embedded in extravascular matter, i.e. the Krogh-Erlang model[18] in conjunction with the two-compartment model by Valabregue et al.[35] An illustration of the capillary network relevant to our modeling is shown in Fig. S5.

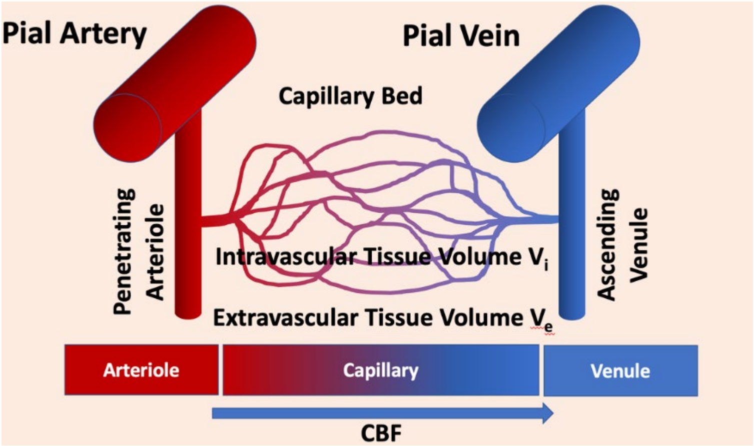

Figure S5. Capillary network and associated oxygen transport (the image is adopted from Kim et al).[51]

O_2 is delivered to the activation region by the blood from the descending arteriole. From the arteriole, the blood is dispersed into a capillary network and is ultimately collected by an ascending venule. O_2 dissolved in the blood plasma is in equilibrium with O_2 bound to hemoglobin, and the equilibrium is described by the Hill equation.[52]

Oxygen transport in and out of the capillary network is modeled by Eq. S1 (also Eq. 1 in the main text).

$$V_i \frac{dC_c(t)}{dt} = CBF(t) \cdot [C_a(t) - C_v(t)] - PS_c \cdot [C_i(t) - C_e(t)] \qquad \text{(S1)}$$

The rate of change in the concentration of oxygen molecules in the capillaries (C_c), including bound and unbound O_2, is given on the left-hand-side (LHS) of Eq. S1. Here V_i denotes the intracapillary volume, which is typically given per 100g of tissue, i.e. mL/100g tissue.

The first term on the right-hand-side of Eq. S1 describes the influx of oxygen into the capillaries, where $CBF(t)$ is the blood flow at time t. C_a and C_v are the arterial and venous concentrations of oxygen, respectively, and they include both bound and unbound O_2. The second term represents the rate of extraction of oxygen from the capillary compartment into extravascular tissue. PS_c is the O_2 mass transfer proportionality parameter that is often written as the product of the capillary O_2 permeability, P [cm/s], and capillary surface area, S_c [cm^2/100g tissue]. Note, however, that the precise microscopic assignment of P and S_c is still debated, and some researchers[49] prefer to define and use another proportionality constant (KTO_2) which does not impose such a strict microscopic interpretation. C_i [μM] and C_e [μM] represent the

volume-averaged concentrations of dissolved (unbound) oxygen in the intravascular and extravascular compartments, respectively.

The extraction rate of oxygen from the capillary is proportional to the concentration gradient between the intravascular and extravascular compartments. Thus, on general grounds, the capillary permeability (P) is expected to be proportional to the oxygen diffusion coefficient and inversely proportional to a characteristic length scale on the order of the capillary radius (but not necessarily equal to it).

A similar equation describes the rate of change in the extravascular oxygen concentration dC_e/dt (also see Eq. 2 in the main text):

$$V_e \frac{dC_e(t)}{dt} = PS_c \cdot [C_i(t) - C_e(t)] - CMRO_2(t) \quad \text{(S2)}$$

where V_e (also defined per 100g of tissue] denotes the extravascular volume, so that $V_e = V_t - V_i$, where V_t is the total tissue volume.[53] In Eq. S2, the net-flow of oxygen includes its diffusion from the intravascular compartment (first term on RHS) and its consumption in tissue (second term on RHS). The tissue oxygen consumption rate is called the Cerebral Metabolic Rate of Oxygen ($CMRO_2$), and it is typically measured in μmole/s/100g tissue. The variables and parameters in Eqs. S1 and S2 are summarized in Table S1.

Table S1. Variables and parameters used in modeling.

Symbol	Unit	Description
V_i	mL / 100g tissue	Capillary (intravascular) volume per unit mass of brain tissue.
V_e	mL / 100g tissue	Extravascular volume per unit mass of brain tissue.
V_t	mL / 100g tissue	Full tissue volume (V_i + V_e) per unit mass of brain tissue.
CBF	mL / s /100g tissue	Cerebral blood flow per unit mass of brain tissue.
C_a	μM	Arterial O_2 concentration (includes both Hb-bound and unbound O_2)
C_c	μM	Capillary O_2 concentration (includes both Hb-bound and unbound O_2)
C_v	μM	Venous O_2 concentration (includes both Hb-bound and unbound O_2)
C_i	μM	Concentration of O_2 dissolved in capillary blood plasma (unbound O_2 only)
C_e	μM	O_2 concentration in the extravascular space of brain tissue.
P	cm / s	O_2 permeability coefficient
S_c	cm^2 / 100g tissue	Capillary surface area per unit mass of brain tissue.
$CMRO_2$	μmol / s / 100g Tissue	Cerebral metabolic rate of O_2

The Krogh-Erlang cylinder model for C_e, $C(\rho)$ and PS_c. The Krogh-Erlang cylinder model (Fig. S6) specifies volume-averaged concentrations C_i and C_e and provides a form for the dependence of oxygen concentration on the the radial distance (ρ), $C(\rho)$. For a single cylinder, the capillary radius is denoted as ρ_1, and the outer edge of the extravascular compartment has radius ρ_2.

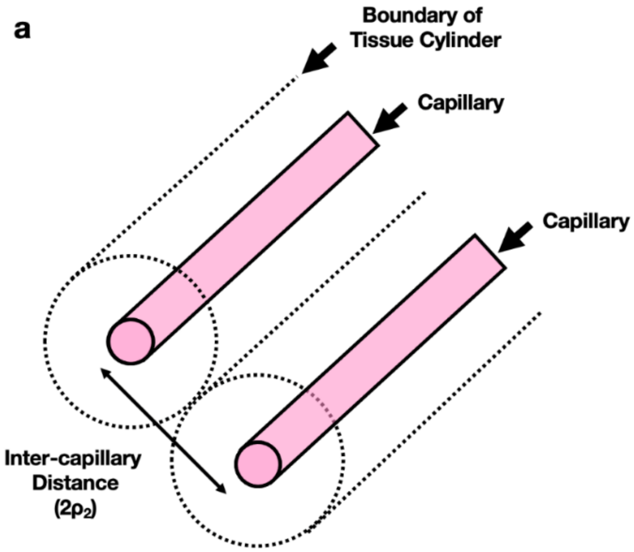

Figure S5. (a) Schematic of the Krogh-Erlang cylinder model. Each cylindrical capillary is surrounded by a coaxial extravascular tissue cylindrical shell. The closest neighboring capillaries are shown placed one inter-capillary distance ($2\rho_2$) away from one another. (b) Cross-section view of a single capillary and the corresponding surrounding extravascular tissue cylindrical shell. Oxygen from the capillary diffuses out to the edge of the extravascular cylinder. The distance from the capillary wall to the tissue boundary is typically set to be one-half of the average inter-capillary distance (ρ_2).

Within this model, many geometric parameters are constrained and related to one another. For example, the fractional volume of capillary in tissue, $V_i/V_t = (\rho_1/\rho_2)^2$, and the total length of the cylinder (for the one cylinder-model) is $L = V_t/\pi(\rho_2)^2$. (Note, in the uniform capillary array version of the model, L represents the sum of the lengths of all capillaries.)

We estimate the oxygen concentration distribution, $C(\rho)$, in the extravascular compartment from the steady-state solution of the time-dependent diffusion equation with loss given as:

$$\frac{\partial C}{\partial t} = D\nabla^2 C - M \qquad \text{(S3)}$$

Here, M is the $CMRO_2$ density in the extravascular volume: $M = CMRO_2/V_e$. The model assumes that M and D are spatially uniform in the extravascular volume. The model also assumes that the intracapillary oxygen concentration is also uniform, which effectively implies that oxygen inside the capillary (including the capillary wall) equilibrates rapidly throughout the volume compared to the equilibration throughout the

extravascular compartment, and that the oxygen transport properties of the capillary wall are the same as those of the capillary interior.

As a boundary condition, we assume that the diffusive O_2 flux at the extravascular outer cylinder boundary is zero: $\left.\frac{dC}{d\rho}\right|_{\rho=\rho_2} = 0$. We also assume that the concentration on the capillary wall edge, $C(\rho_1) = C_i$. The steady-state solution of this problem is given by Krogh[18] and, in more detail, by Popel:[54]

$$C(\rho) = C(\rho_1) + \frac{M}{4D}(\rho^2 - \rho_1^2) - \frac{M\rho_2^2}{2D}\ln\left(\frac{\rho}{\rho_1}\right) \quad \text{(S4)}$$

The volume-averaged extravascular, C_e, is then found by computing the spatial average of $C(\rho)$ over the extravascular compartment volume.

$$C_e = \frac{1}{V_e}\int_{V_e} C(\rho)dV = -\frac{M}{2D}\left(\frac{\rho_2^4 \ln\left(\frac{\rho_2}{\rho_1}\right)}{\rho_2^2 - \rho_1^2}\right) + \frac{M}{8D}(3\rho_2^2 - \rho_1^2) + C(\rho_1) \quad \text{(S5)}$$

An exemplary profile of the partial pressure of oxygen in the extravascular cylinder is shown in Fig. S7. The volume-averaged pO_2 is also indicated.

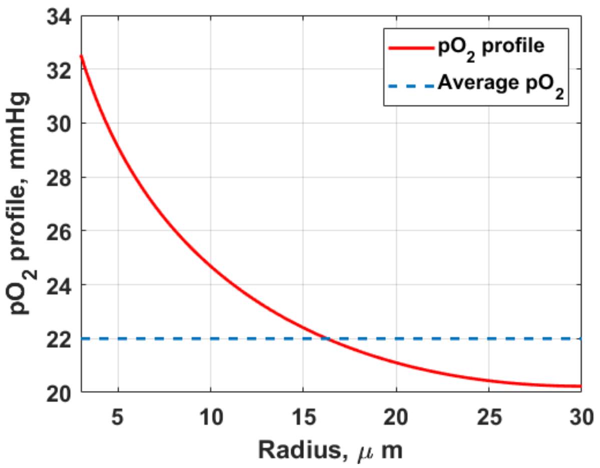

Figure S7. Oxygen partial pressure (pO_2) profile along the radial distance from the wall (ρ_1=3 μm) to the outer surface of the tissue cylinder (ρ_2=30 μm) in the Krogh cylinder model. $CMRO_2$ and D are set to be 3.4 μmol/s/100g tissue and 1.7×10^{-5} cm^2/s respectively. The volume-averaged pO_2 is 22 mmHg.

From Eq. S5, we can see that one parameter that we experimentally measure, C_e, is predicted to be an explicit function of morphological geometric factors ρ_1 and ρ_2, as well as of the physiological factors M and D. For fixed capillary volume fraction and capillary radius, the average O_2 concentration is thus a function of M and D.

It is useful to rewrite Eq. S5 in other ways. For example, the expression below reveals linear relationship between M and the oxygen concentration difference between the compartments.

$$M = \frac{D}{F(\rho_1,\rho_2)}[C(\rho_1) - C_e]; \qquad F(\rho_1,\rho_2) = \frac{\rho_2^4 \ln\left(\frac{\rho_2}{\rho_1}\right)}{2(\rho_2^2-\rho_1^2)} - \frac{3\rho_2^2-\rho_1^2}{8} \tag{S6}$$

If we substitute $C(\rho_1)$ for C_i, and replace M with $CMRO_2/V_e$ in Eq. S6, we obtain an expression for the $CMRO_2$ in a steady-state in two-compartment model (Eq. S7).

$$CMRO_2 = PS_c(C_i - C_e) \tag{S7}$$

Equation S7 relates the experimentally measured paramerers, *i.e.,* C_i and C_e, to $CMRO_2$ and PS_c. Importantly, PS_c depends on microvascular parameters that have been previously measured or estimated, *i.e.,* ρ_1, ρ_2, D, and L. (Note, L is a function of V_t and ρ_2).

Notably, the Krogh-based model gives an explicit formula for PS_c:

$$PS_c = \frac{2\pi LD}{G\left(\frac{\rho_2}{\rho_1}\right)}; \qquad G\left(\frac{\rho_2}{\rho_1}\right) = \frac{\left(\frac{\rho_2}{\rho_1}\right)^4 \ln\left(\frac{\rho_2}{\rho_1}\right)}{\left[\left(\frac{\rho_2}{\rho_1}\right)^2 - 1\right]^2} - \frac{3\left(\frac{\rho_2}{\rho_1}\right)^2 - 1}{4\left[\left(\frac{\rho_2}{\rho_1}\right)^2 - 1\right]} \tag{S8}$$

The function $G(\rho_2/\rho_1)$ in Eq. S8 is a function of the ratio of inter-capillary distance to capillary diameter. Therefore, with caveats related to the approximations/simplifications outlined above, PS_c represents the oxygen mass transfer coefficient connecting $CMRO_2$ to the oxygen gradient across intra- and extravascular tissue compartments. Moreover, within this model, P and S_c can be separately defined. For example, if we take S_c to be the capillary surface area, *i.e.,* $S_c = 2\pi\rho_1 L$, then we generate an oxygen permeability associated with tissues surrounding the capillary wall, *i.e.,* $P = D/[\rho_1 G(\rho_2/\rho_1)]$. The function, $G(\rho_2/\rho_1)$, is not significantly larger than unity for typical microvascular parameters, *e.g.,* it

ranges from ~1.1 to ~1.6 for typical capillary diameter and separation when V_i/V_t is between 1 and 3 percent.

The Krogh cylinder-based model also provides an explicit formula for the extravascular compartment exponential relaxation time constant, $\tau = V_e/PS_c$, in terms of ρ_1, ρ_2 and D. This time constant arises naturally in Eq. S2 and can be important for the description of metabolism dynamics at early times. Interestingly, the relationship between τ and the mean time, Δt, for oxygen to diffuse the distance ρ_2 is well defined in the model; the ratio $\tau/\Delta t$ ranges from approximately 2.1 to 3.2 for typical system parameters, and depends only weakly on ρ_2/ρ_1 in the physiologically relevant experimental regime (*i.e.,* V_i/V_t between 1 and 3 percent). Thus, the time for the extravascular compartment to reach a new steady-state following an impulse change in oxygen concentration is 2x-3x longer than the mean time it takes for oxygen to diffuse to the extravascular tissue boundary, ρ_2.

Limitations of the model. As many biophysical models which give exact results, the present model has important limitations. For example, besides the specific numerical approximations noted above, the model ignores the heterogeneous nature of the capillary network. In practice, the capillary size and especially the inter-capillary separation is not uniform. A more complex theory should average over the distribution of regions with different morphologies. In that case, even if we continue to adopt the core cylinder model, a slightly more complex theory would quantitatively predict a distribution of PS_c and τ. Accounting for these distributions could change the predicted dynamics, *i.e.,* compared to replacing distributions by their average values.

Another limitation derives from the fact that tissue has both neurons and other cells, and they likely have different temporal metabolic responses to stimulation. Still another limitation is the description of the capillary wall as a uniform extension of the inner capillary. For example, the oxygen diffusion coefficient in the capillary wall is likely to be different from that in the surrounding space. All of these factors need to be considered in data interpretation. Notably, our new methodology will make confrontation of these issues possible.

9. Selection of PS_c

Eq. S2 enables computation of $CMRO_2$, however the computed profiles are dependent on the choice of parameters V_e and PS_c. The volume V_e is known from experiments.[41, 42, 53] However, the value of PS_c, which affects both $CMRO_2$ magnitude and dynamics, is not well-known, which reflects the fact that

measurements of oxygen diffusion coefficient in tissue us notoriously difficult. PS_c values have been measured,[55] computed,[35, 49, 56] and derived by combining measurements and calculations.[57, 58, 59, 60] According to a recent review,[49] the reported value of PS_c vary in the range of 35-230 mL/s/100g tissue. Below we describe how we chose PS_c from the available literature data, including teh reported measurements of morphological and physiological parameters.

According to Eq. S8, the relevant morphological parameters are the capillary radius (ρ_1), one-half the inter-capillary separation (ρ_2) and the volume fraction of intravascular tissue (V_i/V_t). These parameters also determine L and V_e. Reported values of ρ_1 vary from 2 to 3.5 µm.[60-64] Reported values of ρ_2 vary from 10 to 30 µm, corresponding to average inter-capillary distances ranging from 20 to 60 µm.[39, 61, 65] Reported intravascular volume fraction, V_i/V_t, in tissue ranges from 1% to 3%.[41, 61, 62, 64, 66] Tables S2-S4 summarize the range of possible morphological parameters (ρ_1, ρ_2, and L) for V_i/V_t equal to 0.01, 0.02, and 0.03. Note, all of these reported morphological parameters were measured in rats.

Table S2. Radii of capillary (ρ_1), Radii of tissue cylinder (ρ_2) and corresponding lengths are tabulated under the constraint of V_i/V_t = 1%.

ρ_1 (µm)	2.0	2.5	3.0
ρ_2 (υm)	20	25	30
L (km)	75.8	48.5	33.7

Table S3. Radii of capillary (ρ_1), Radii of tissue cylinder (ρ_2) and corresponding lengths are tabulated under the constraint of V_i/V_t = 2%.

ρ_1 (µm)	2.0	2.5	3.0	3.5
ρ_2 (µm)	14	18	21	25
L (km)	152	97.0	67.4	49.5

Table S4. Radii of capillary (ρ_1), Radii of tissue cylinder (ρ_2) and corresponding lengths are tabulated under the constraint of V_i/V_t = 3%.

ρ_1 (µm)	2.0	2.5	3.0	3.5
ρ_2 (µm)	12	14	17	20
L (km)	227	146	101	74.2

The other relevant physiological parameters are the oxygen diffusion coefficient, D, and the *baseline* (normal) $CMRO_2$. Reported values of D vary from 1.4 to 1.9 [$\times 10^{-5}$ cm^2/s] at 37°C.[67-70] Note, in the cited papers objects varied from small rodents to a human brain tissue. The reported baseline $CMRO_2$ of rat brain ranges from 3 to 7.4 [μmol/s/100g tissue].[44, 45, 71, 72]

The cylindrical model for capillary and surrounding tissue enables determination of PS_c based on the full range of reported ρ_1, ρ_2, V_i/V_t, and D and using Eq. S8. The resulting "allowed" variation of PS_c is large, spanning from 137 to 2470 [mL/s/100g tissue]. Tables S5-S7 show the values of PS_c (in **bold**) as a function of ρ_1, ρ_2, V_i/V_t, and D; note ρ_2 is fixed by choice of ρ_1 and V_i/V_t. This potential variation of PS_c is much larger than reported in the literature.

Table S5. PS_c (bold), $CMRO_2$ vs D, ρ_1, ρ_2 for intravascular volume fraction, V_i/V_t, of 0.01 (1%). PS_c values [mL/s/100g tissue] computed using Eq. S8 as a function of D [10^{-5} cm^2/s], ρ_1 and ρ_2 [μm]. Baseline $CMRO_2$ values [μmol/s/100g tissue] are computed according to Eq. S7 using PS_c based on different values of D [10^{-5} cm^2/s] and ρ_1 [μm]. The baseline $CMRO_2$ values that are consistent with the range of prior measurements are shown in green-shaded cells.

ρ_1 / ρ_2 / D	2.0<br>20	2.5<br>25	3.0<br>30
1.4	**418**, 7.1	**268**, 4.5	**186**, 3.2
1.5	**448**, 7.6	**287**, 4.9	**199**, 3.4
1.6	**478**, 8.1	**306**, 5.2	**212**, 3.6
1.7	**508**, 9.1	**325**, 5.5	**226**, 3.8
1.8	**538**, 9.6	**344**, 5.8	**239**, 4.1
1.9	**568**, 9.6	**363**, 6.2	**252**, 4.3

Table S6. PS_c (bold), $CMRO_2$ vs D, ρ_1, ρ_2 for intravascular volume fraction of 0.02 (2%). PS_c values [mL/s/100g tissue] computed from Eq. S8 as a function of D [10^{-5} cm^2/sec], ρ_1 and ρ_2 [μm]. Baseline $CMRO_2$ values [μmol/s/100g tissue] are computed according to Eq. S7, using the PS_c based on different values of D [10^{-5} cm^2/s] and ρ_1 [μm]. The baseline $CMRO_2$ values that are consistent with the range of prior measurements are shown in green-shaded cells.

ρ_1 / ρ_2 / D	2.0<br>14	2.5<br>18	3.0<br>25	3.5<br>28
1.4	**1040**, 17.9	**669**, 11.5	**464**, 8.0	**341**, 5.8
1.5	**1120**, 19.2	**716**, 12.3	**497**, 8.5	**365**, 6.3
1.6	**1190**, 20.5	**764**, 13.1	**531**, 9.1	**390**, 6.7
1.7	**1270**, 21.7	**812**, 13.9	**564**, 9.7	**414**, 7.1
1.8	**1340**, 23.0	**860**, 14.7	**597**, 10.2	**439**, 7.5
1.9	**1420**, 24.3	**907**, 15.5	**630**, 10.8	**463**, 7.9

Table S7. PS_c (Bold), $CMRO_2$ vs D, ρ_1, ρ_2 for intravascular volume fraction of 0.03 (3%). PS_c values [mL/sec/100g Tissue] computed from Eq. S8 as a function of D [10^{-5} cm^2/sec], ρ_1 and ρ_2 [μm]. Baseline $CMRO_2$ values [μmoles/sec/100g Tissue] are computed according to Eq. S7, using the PS_c based on different values of D [10^{-5} cm^2/sec] and ρ_1 [μm]. The baseline $CMRO_2$ values that are consistent with the range of prior measurements are shown in green-shaded cells.

ρ_1 / ρ_2 / D	2.0<br>12	2.5<br>14	3.0<br>17	3.5<br>20
1.4	**1820**, 31.5	**1170**, 20.2	**810**, 14.0	**595**, 10.3
1.5	**1950**, 33.8	**1250**, 21.6	**867**, 15.0	**637**, 11.0
1.6	**2080**, 36.0	**1330**, 23.1	**925**, 16.0	**680**, 11.8
1.7	**2210**, 38.3	**1420**, 24.5	**983**, 17.0	**722**, 12.5
1.8	**2340**, 40.6	**1500**, 26.0	**1040**, 18.0	**765**, 13.2
1.9	**2470**, 42.8	**1580**, 27.4	**1110**, 19.0	**807**, 14.0

To further constrain PS_c, we next utilize prior measurements of baseline (normal) $CMRO_2$ and our measurements of intravascular and extravascular pO_2. Specifically, our measurements found baseline p_iO_2=33.5 mmHg and p_eO_2=21.3 mmHg. Using the reported range of baseline $CMRO_2$ and Eq. S7, *i.e.,* $CMRO_2 = PS_c(C_i - C_e)$, we identify a much smaller subset of PS_c's, which satisfy Eq. S7 subject to constraints of tissue morphology and oxygen diffusion. These results are summarized in Tables S5-S7, which show the predicted $CMRO_2$ as a function of ρ_1, ρ_2, V_i/V_t, and D for the cases where 3 μmol/s< $CMRO_2$ < 7.4 μmol/s. The **green shaded rectangles** in the Tables correspond to the allowed values of $CMRO_2$. Note, PS_c is given in bold next to $CMRO_2$ in these green shaded rectangles.

Taken together, our modeling and analysis suggest that PS_c has a value constrained approximately to the range from 180 to 430 [mL/s/100g tissue]. The choice of PS_c amongst this relatively large range of possibilities has consequences for the magnitude and dynamics of response of $CMRO_2$. For our calculations we choose PS_c = 210 [mL/s/100g tissue]. In making this selection, we used morphological and physiological parameters towards the middle of the reported ranges; for example, D $\cong 1.6\times10^{-5}$ cm^2/s and ρ_1 = 3.0 μm and ρ_2 = 30 μm. Note also, this selection of 210 [mL/s/100g tissue] is similar to the value of PS_c found in the only study of rat brain[56] that had a pO_2 (~25 mmHg) comparable to our experiments (~21 mmHg). Lastly, this choice has the virtue that it is also within the range used in all prior studies, which suggest that PS_c has a value between 35 and 230 [mL/s/100g tissue].[49] As a cautionary note, however, a small change of ρ_1 and ρ_2 to 2.5 μm and 25 μm, respectively, gives PS_c ~300 [mL/sec/100g tissue]. Ultimately, to ameliorate this problem it will be valuable to carry out more experiments to further constrain the possible values of PS_c.

10. Timing analysis

To analyze the dynamics of stimulation-induced traces of CBF, p_iO_2, p_eO_2 and $CMRO_2$, six time-points were sought: a "trigger" time ($t_{trigger}$), the 20%, 50% and 90% rise-time from baseline ($t_{20\%}$, $t_{50\%}$, $t_{90\%}$), the time at-peak (t_{peak}), and the 50% fall-time from peak (t_{fall}). To obtain these time points we averaged the 13 single-stimulation traces. Further, to reduce noise in the single-shot stimulation data, we smoothed each single-shot raw data trace utilizing a low-pass-filter de-noising technique. The low-pass filtering kernel was a sliding Gaussian function with a full-width-at-half-maximum of 0.3 s (twice the measurement time-step). After application of the filter, we computed a mean trace from the 13 responses as shown in Fig. 4 in the main text. We also computed a trace of the standard error (standard deviation of the mean) for additional temporal error analysis. Exemplary raw and de-noised data are shown in Fig. S8.

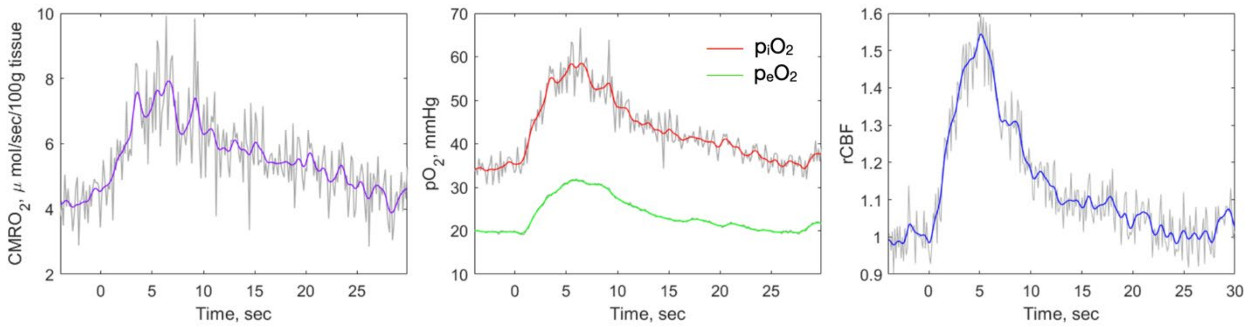

Figure S8. De-noised single-stimulation traces. Each trace is low-pass filtered (FWHM=300 ms). In each plot, the raw data is marked in gray. Notice, the p_eO_2 trace is nearly noise-free compared to other traces.

For temporal analysis we adopted an approach published previously.[43] The de-noised average data were normalized, so that the baseline is set to 0, and the peak corresponds to 1 (Fig. 4, main text). The standard error (standard deviation of the mean) is accordingly normalized, *i.e.*, divided by the peak magnitude. Our determination of the trigger time/height sought to identify a peak height that was clearly larger than measurement noise. To this end we first computed the standard deviation (σ) of full-dynamic $\mathrm{CMRO_2}$ during the 4s-long baseline period prior to activation. We chose full-dynamic $\mathrm{CMRO_2}$ for this task because it was our noisiest signal. The resultant baseline standard deviation was $\sigma = 0.03$. We then defined the trigger time/height of full-dynamic $\mathrm{CMRO_2}$ to be the point on the trace where the signal had definitively increased (out of the noise) to $3\sigma = 0.09$. Thus, in practice, $t_{trigger}$ is determined from the 9% rise time-point with respect to the baseline ($t_{9\%}$) for full-dynamic $\mathrm{CMRO_2}$ and all other traces. Note, the term trigger applies directly to $\mathrm{CMRO_2}$, CBF, and p_iO_2 traces but not p_eO_2. Determination of $t_{20\%}$, $t_{50\%}$, $t_{90\%}$, t_{peak}, t_{fall}, and $t_{trigger}= t_{9\%}$ was straightforward. For example, $t_{20\%}$ is determined by the intersection of normalized trace with the horizontal line displaced vertically by 0.2 from the horizontal axis. We assigned the timing uncertainties following a standard approach.[73] The results of the timing analysis are summarized in Table S8.

Table S8. Timing of $t_{trigger} = t_{9\%}$, $t_{20\%}$, $t_{90\%}$, t_{peak}, and t_{fall}, corresponding to the 9%, 20%, 90% rise during functional activation, and the peak-time and 50% fall-time, respectively. Estimates are given for CBF, pO_2 and $CMRO_2$ (both full- and truncated-dynamic models). The confidence interval for the timing was computed from standard error of the mean of the set of 13 traces (in parenthesis).

Parameters	$t_{9\%}$, sec	$t_{20\%}$, sec	$t_{50\%}$, sec	$t_{90\%}$, sec	t_{peak}, sec	t_{fall}, sec
CBF	1.0 (0.2)	1.4 (0.1)	2.1 (0.1)	3.5 (0.3)	5.0 (0.4)	7.5 (0.2)
p_iO_2	1.3 (0.1)	1.7 (0.1)	2.6 (0.1)	4.4 (0.1)	5.7 (0.2)	11.1 (0.3)
p_eO_2	-	2.1 (0.1)	3.1 (0.1)	5.2 (0.3)	6.5 (1.5)	11.1 (0.4)
$fCMRO_2$	0.2 (0.2)	1.6 (0.2)	2.4 (0.2)	3.6 (0.6)	4.5 (0.2)	11.5 (0.5)
$tCMRO_2$	0.8 (0.3)	1.2 (0.2)	2.1 (0.1)	3.5 (0.2)	4.5 (0.2)	10.5 (0.7)

11. Records of CBF and ABP during stimulation

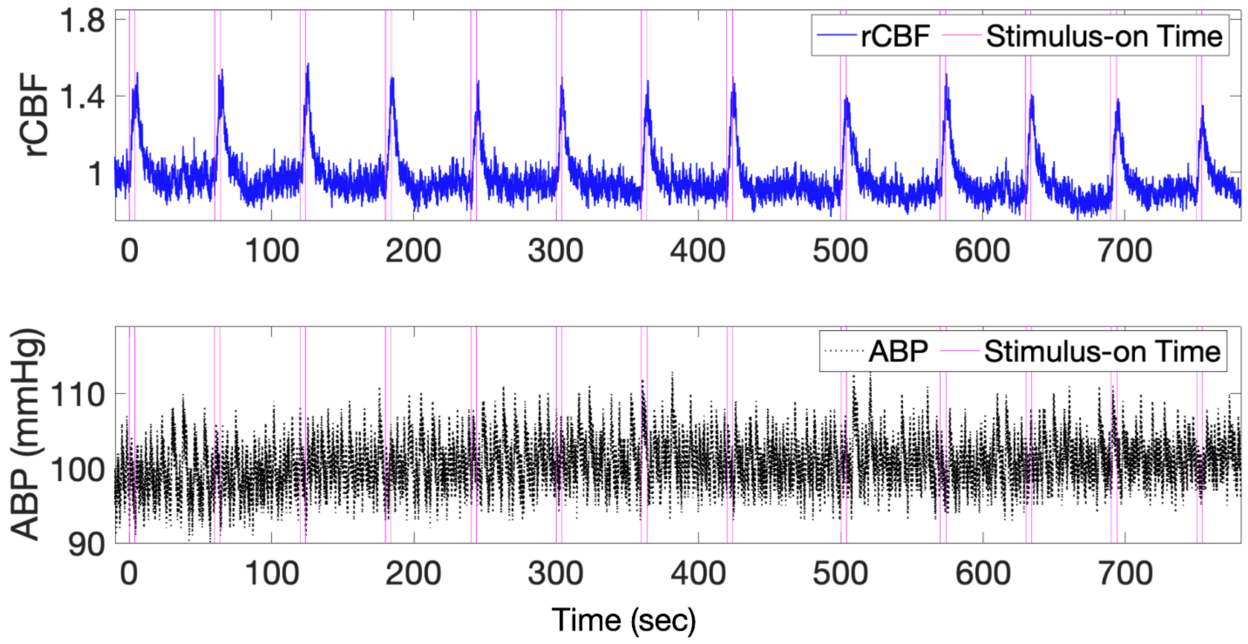

Figure S9. Concurrent records relative CBF (rCBF) and arterial blood pressure (ABP) in a representative experiment.

12. Potential measurement artifacts: oxygen consumption, probe distribution

Potentially, oxygen measurements by phosphorescence quenching can lead to oxygen consumption. The consumption is not due to the probe per se, but rather to scavenging of singlet oxygen, a by-product of the triplet quenching reaction, by other organic molecules in the medium. Singlet oxygen is indiscriminate in choosing its substrate, and the probe is certainly one of its potential targets. However, the probe is usually present in micromolar concentrations, while the "concentration" of other organic molecules in tissue (lipids, proteins etc) is in molar range. Hence, virtually all singlet oxygen that gets scavenged, is scavenged by biological matter.

Nevertheless, it is important to realize that the lifetime of singlet oxygen in water is only ~3 μs. Thus, most of the singlet oxygen generated in blood plasma or interstitial space, decays back to the ground state (triplet O_2) faster than it interacts with organic molecules. Only at very high light fluxes, high probe concentrations and in systems with limited supply of oxygen one might observe oxygen depletion upon phosphorescence quenching measurements. It was clearly not the case in the experiments reported in this work. We performed control measurements where a solution of the probe (Oxyphor PtG4, 2 μM) in 1M glycerol, 5mM glucose, 5mM glutathione (GSH), and 10mM HEPES (pH 7.2) and ~1 mM bovine serum albumin (BSA) - a solution mimicking cellular milieu, - in a sealed vial was exposed to the same measurements as in or *in vivo* experiments, showing that oxygen consumption was not a concern.

Probe concentration does not affect phosphorescence lifetime measurements per se, but it does affect the signal-to-noise ratio. In a highly heterogeneous environment, such as tissue, it is possible that within the volume sampled by light more of the probe would somehow end up in a more oxygenated region *vs* less oxygenated region or *vice versa*, i.e. a situation would occur where a probe gradient would affect the average measured pO_2 value. However, such a distribution would occur randomly, and since in our experiments the sampled volume was rather large (~1 mm^3), it is likely that such microheterogeneities would average out. To verify that it was indeed the case, in our experiments we often performed controlled measurements where the position of the illuminated spot on the brain surface was slightly changed. The pO_2 values remained very similar, indicating that the heterogeneity of the probe distribution had only a minimal effect on the sampled traces.

13. NMR spectra

CBzNH-AG¹(GluO*ᵗ*Bu)₂: ¹H NMR

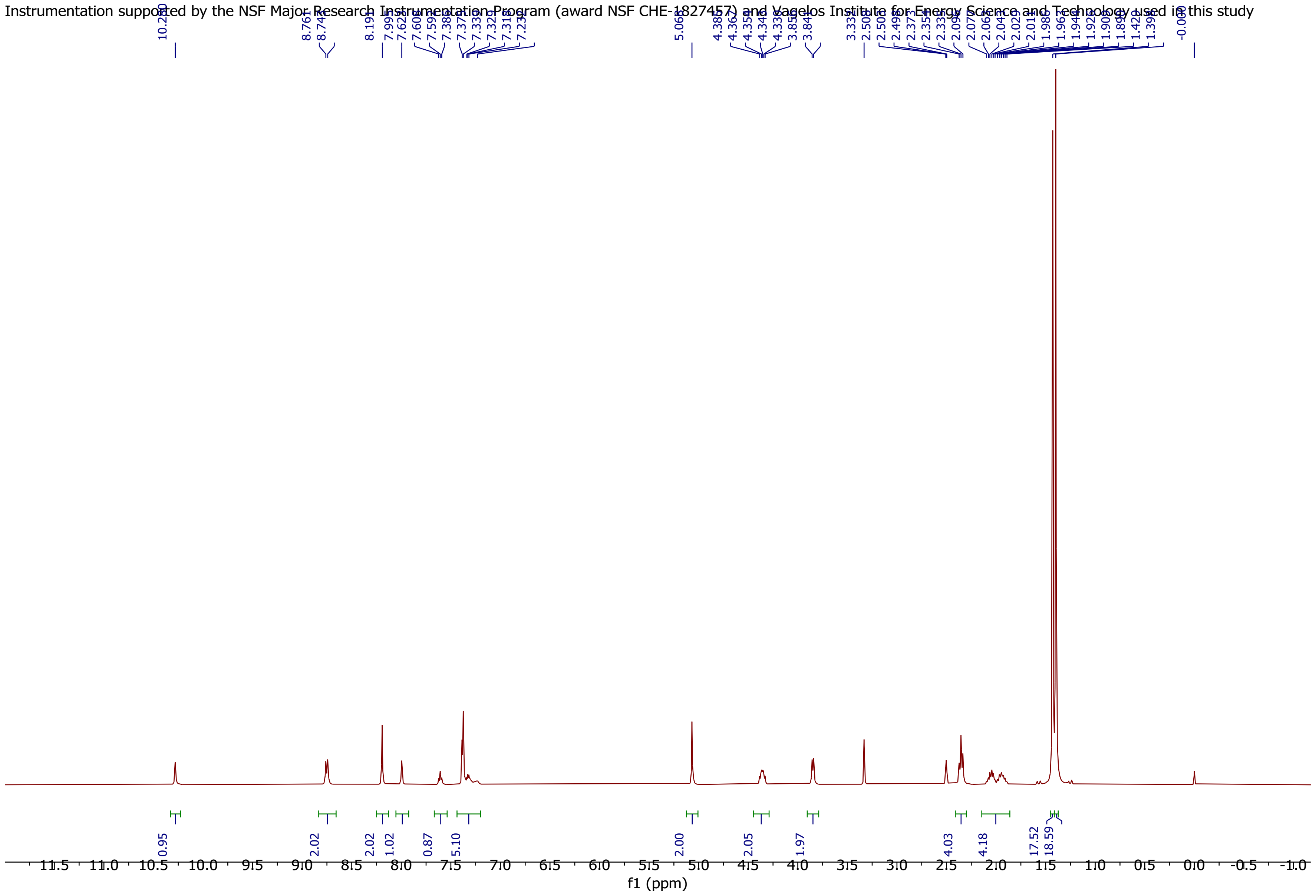

CBzNH-AG¹(GluO*ᵗ*Bu)₂: ^{13}C NMR

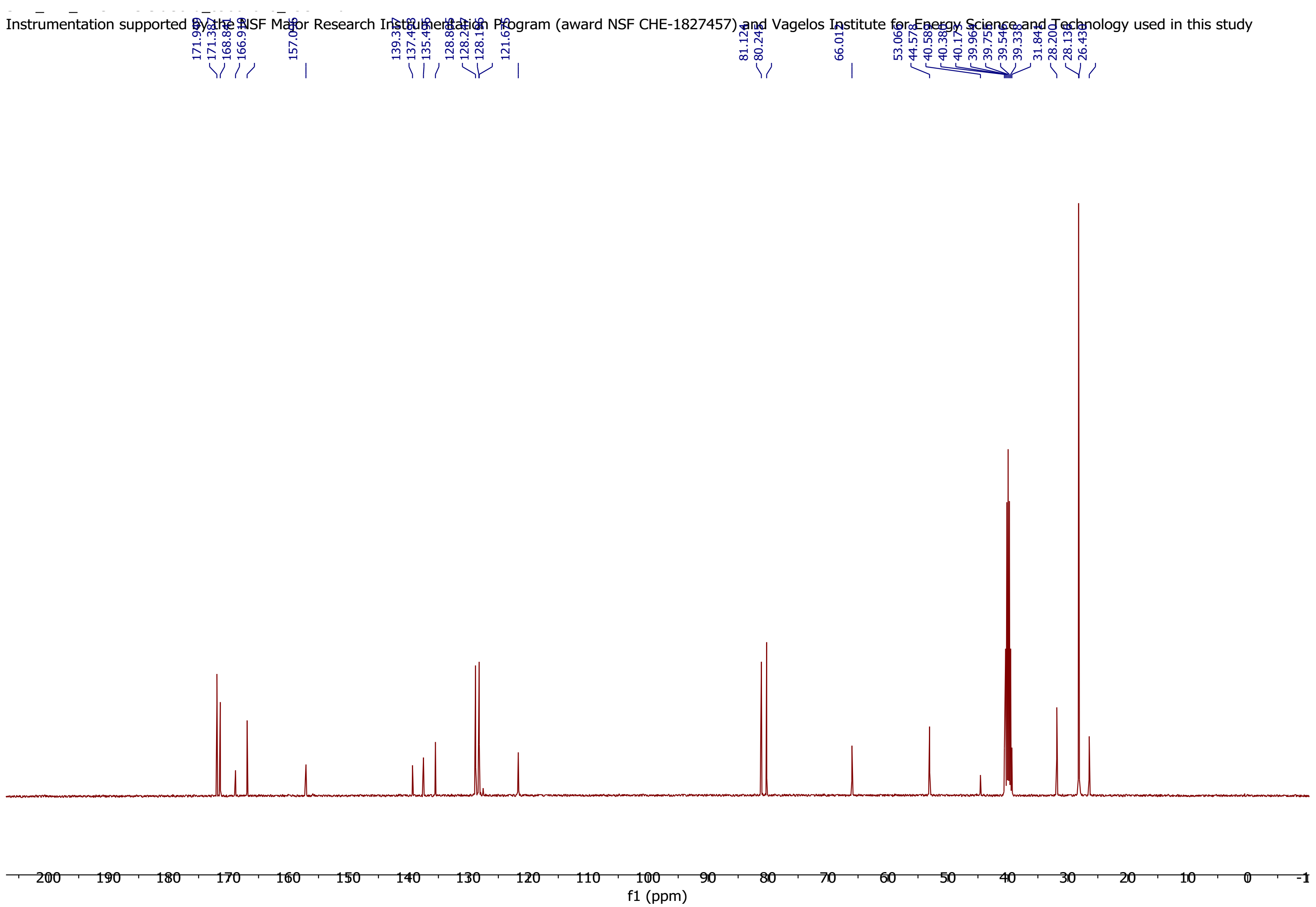

NH_2-AG[1](GluO^{t}Bu)$_2$: ^{1}H NMR

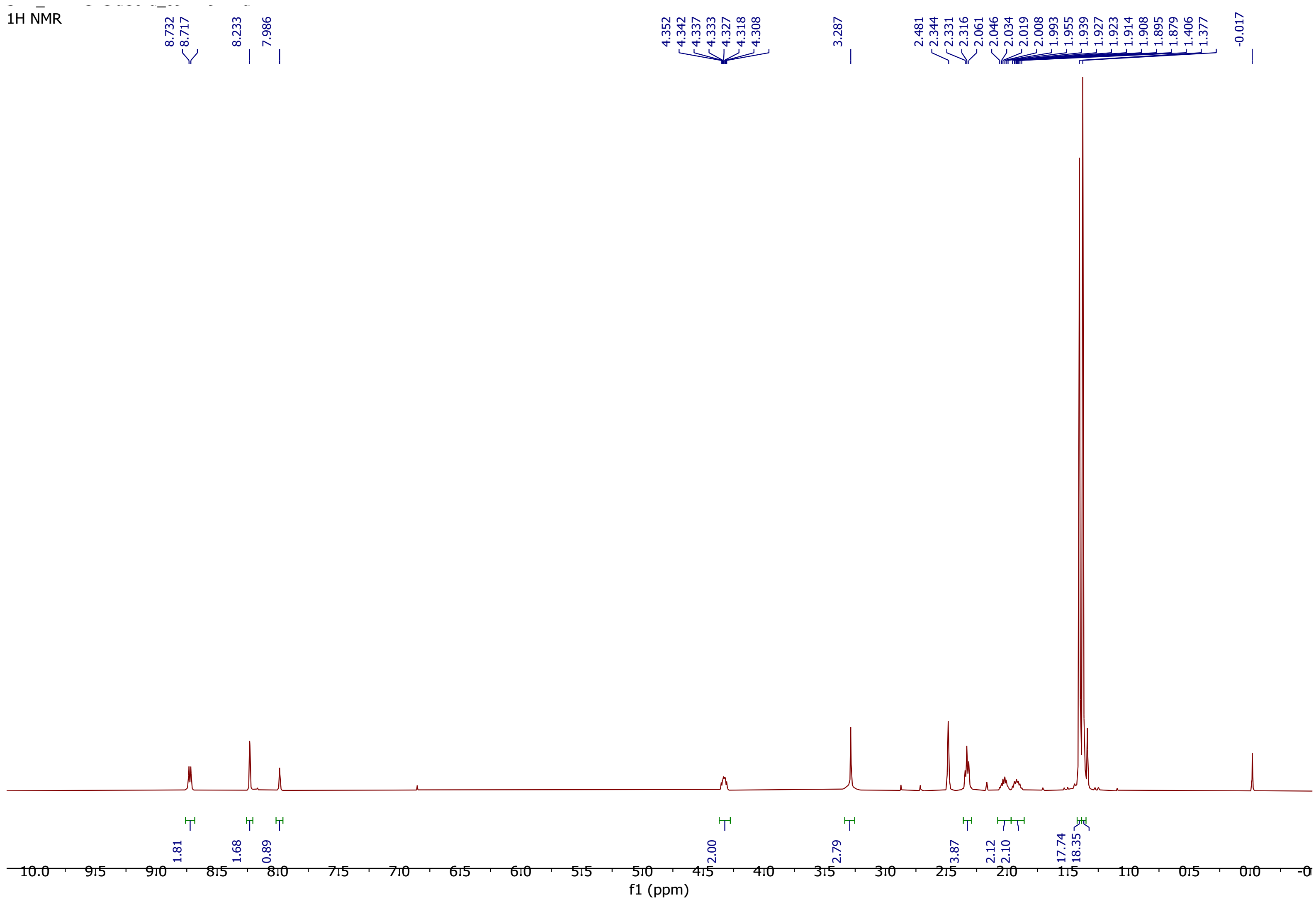

NH$_2$-AG^1(GluO^{t}Bu)$_2$: ^{13}C NMR

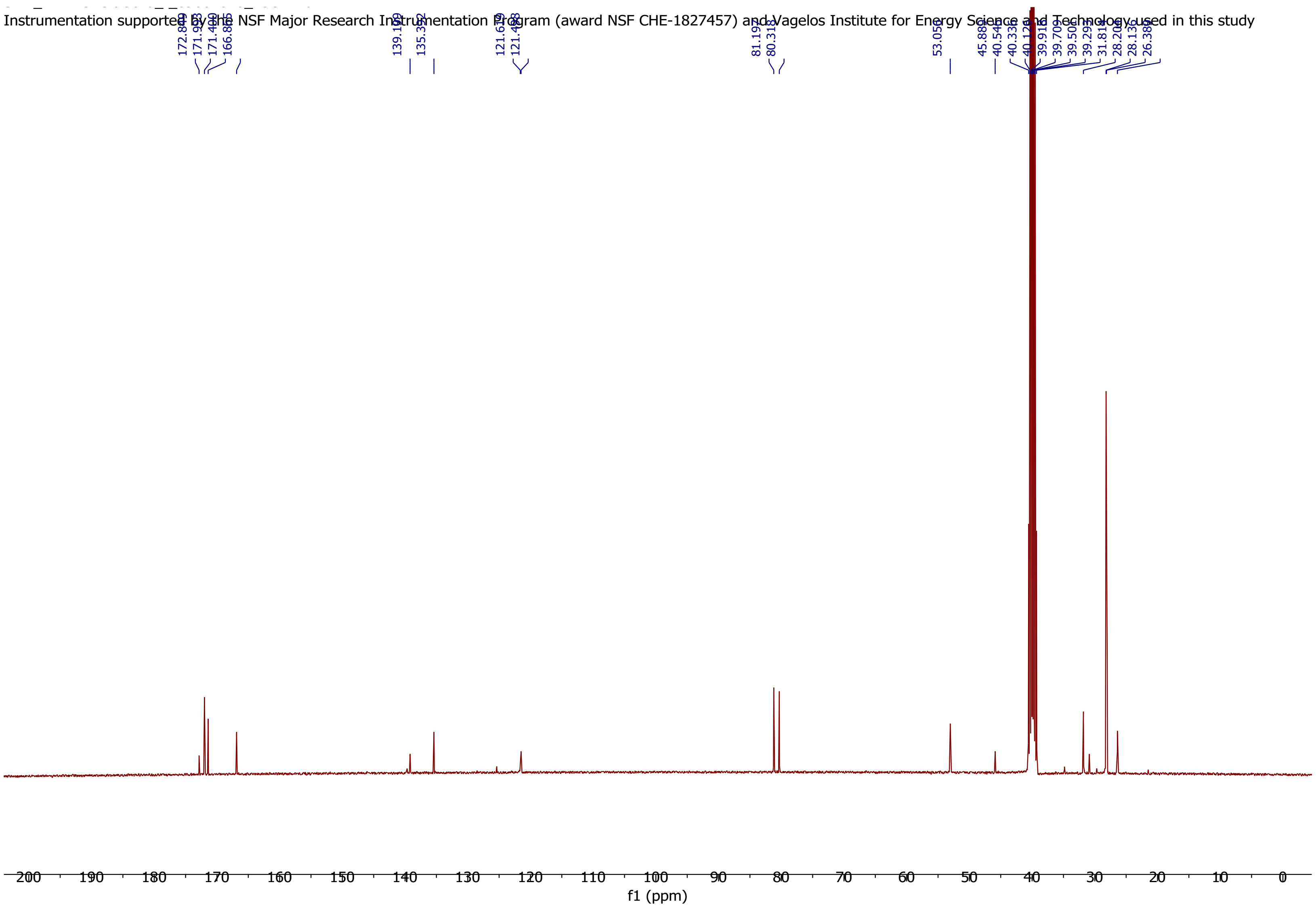

PtTPP-(AG¹GluO*ᵗ*Bu)₈: ¹H NMR

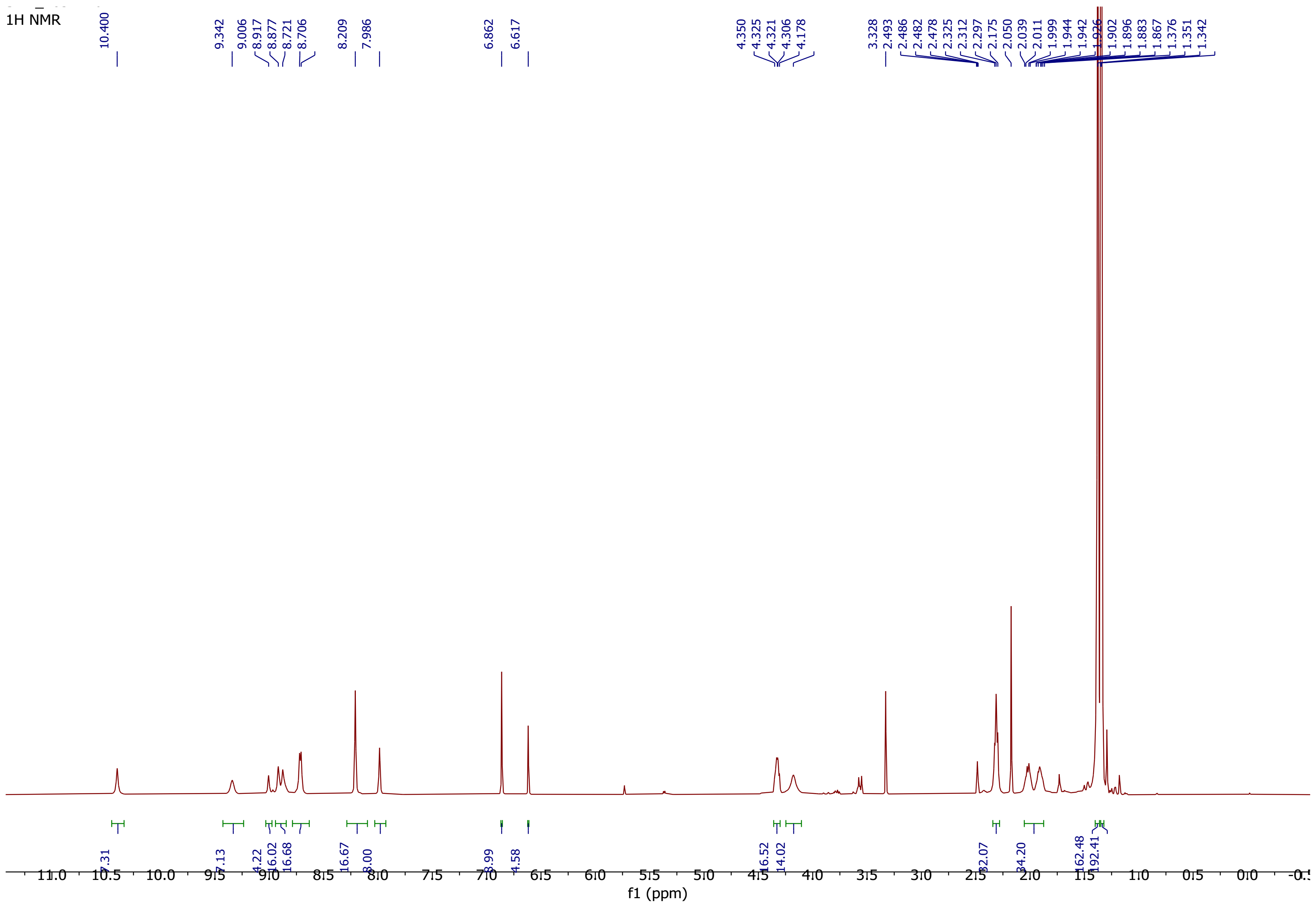

Supplement: Supplementary file 1 [file NPh_009_045006_SD001.pdf]
